# Suppression of inflammation-induced lung cancer cells proliferation and metastasis by exiguaflavanone A and exiguaflavanone B from *Sophora exigua* root extract through NLRP3 inflammasome pathway inhibition

**DOI:** 10.3389/fphar.2023.1243727

**Published:** 2023-11-10

**Authors:** Punnida Arjsri, Kamonwan Srisawad, Warathit Semmarath, Sonthaya Umsumarng, Lapamas Rueankham, Aroonchai Saiai, Methee Rungrojsakul, Trinnakorn Katekunlaphan, Songyot Anuchapreeda, Pornngarm Dejkriengkraikul

**Affiliations:** ^1^ Department of Biochemistry, Faculty Medicine, Chiang Mai University, Chiang Mai, Thailand; ^2^ Anticarcinogenesis and Apoptosis Research Cluster, Faculty of Medicine, Chiang Mai University, Chiang Mai, Thailand; ^3^ Akkhraratchakumari Veterinary College, Walailak University, Nakhon Si Thammarat, Thailand; ^4^ Center for Research and Development of Natural Products for Health, Chiang Mai University, Chiang Mai, Thailand; ^5^ Division of Veterinary Preclinical Sciences, Department of Veterinary Biosciences and Veterinary Public Health, Faculty of Veterinary Medicine, Chiang Mai University, Chiang Mai, Thailand; ^6^ Department of Medical Technology, Faculty of Associated Medical Sciences, Chiang Mai University, Chiang Mai, Thailand; ^7^ Department of Chemistry, Faculty of Science, Chiang Mai University, Chiang Mai, Thailand; ^8^ Department of Traditional Chinese Medicine, Faculty of Science, Chandrakasem Rajabhat University, Bangkok, Thailand; ^9^ Department of Chemistry, Faculty of Science, Chandrakasem Rajabhat University, Bangkok, Thailand

**Keywords:** *Sophora exigua*, exiguaflavanone, non-small cell lung cancer, cancer progression and metastasis, NLRP3 inflammasome pathway

## Abstract

**Objective:** Non-small cell lung cancer (NSCLC) is recognized for its aggressive nature and propensity for high rates of metastasis. The NLRP3 inflammasome pathway plays a vital role in the progression of NSCLC. This study aimed to investigate the effects of *S. exigua* extract and its active compounds on NLRP3 regulation in NSCLC using an *in vitro* model.

**Methods:** *S. exigua* was extracted using hexane, ethyl acetate and ethanol to obtain *S. exigua* hexane fraction (SE-Hex), *S. exigua* ethyl acetate fraction (SE-EA), and *S. exigua* ethanol fraction (SE-EtOH) respectively. The active compounds were identified using column chromatography and NMR analysis. A549 cells were primed with lipopolysaccharide (LPS) and adenosine triphosphate (ATP) for activated NLRP3 inflammasome. The anti-inflammatory properties were determined using ELISA assay. The anti-proliferation and anti-metastasis properties against LPS-ATP-induced A549 cells were determined by colony formation, cell cycle, wound healing, and trans-well migration and invasion assays. The inflammatory gene expressions and molecular mechanism were determined using RT-qPCR and Western blot analysis, respectively.

**Results:** SE-EA exhibited the greatest anti-inflammation properties compared with other two fractions as evidenced by the significant inhibition of IL-1β, IL-18, and IL-6, cytokine productions from LPS-ATP-induced A549 cells in a dose-dependent manner (*p* < 0.05). The analysis of active compounds revealed exiguaflavanone A (EGF-A) and exiguaflavanone B (EGF-B) as the major compounds present in SE-EA. Then, SE-EA and its major compound were investigated for the anti-proliferation and anti-metastasis properties. It was found that SE-EA, EGF-A, and EGF-B could inhibit the proliferation of LPS-ATP-induced A549 cells through cell cycle arrest induction at the G0/G1 phase and reducing the expression of cell cycle regulator proteins. Furthermore, SE-EA and its major compounds dose-dependently suppressed migration and invasion of LPS-ATP-induced A549 cells. At the molecular level, SE-EA, EGF-A, and EGF-B significantly downregulated the mRNA expression of IL-1β, IL-18, IL-6, and NLRP3 in LPS-ATP-induced A549 cells. Regarding the mechanistic study, SE-EA, EGF-A, and EGF-B inhibited NLRP3 inflammasome activation through suppressing NLRP3, ASC, pro-caspase-1(p50 form), and cleaved-caspase-1(p20 form) expressions.

**Conclusion:** Targeting NLRP3 inflammasome pathway holds promise as a therapeutic approach to counteract pro-tumorigenic inflammation and develop novel treatments for NSCLC.

## Introduction

Lung cancer is a major global health concern, being the second most prevalent form of cancer and the primary contributor to cancer-related fatalities on a global scale (Huang et al., 2022). It includes different subcategories, wherein non-small cell lung cancer (NSCLC) makes up the majority, representing approximately 85% of all cases (Alduais et al., 2023). Within NSCLC, there are several subtypes such as adenocarcinoma, squamous cell carcinoma, and large cell carcinoma, among others. However, the existing therapies for NSCLC, which include surgery, chemotherapy, and radiotherapy, are inadequate in effectively reducing the elevated mortality rates associated with the disease (Wu and Lin, 2022). Despite advancements in early detection and treatment of NSCLC, the prognosis for patients with this condition remains unfavorable, as evidenced by a less than 20% 5-year overall survival rate (Zappa and Mousa, 2016). The recurrence of tumors and the spread of metastases play a significant role in the unfavorable outcomes and serve as the primary factors contributing to mortality in NSCLC cases (Ko et al., 2021). Hence, acquiring a thorough comprehension of the cellular and molecular mechanisms that trigger and advance NSCLC is of utmost significance.

In recent times, alongside surgery, chemotherapy, and radiotherapy, there has been increasing attention on adjuvant therapies that focus on targeting the tumor microenvironment. A growing body of evidence suggests that chronic inflammation plays a pivotal role in the progression of cancer (Gomes et al., 2014; Stares et al., 2022). While acute inflammation serves as a protective mechanism against infectious pathogens, chronic inflammation is linked to DNA damage and tissue dysfunction, encompassing genetic and epigenetic alterations that contribute to the initiation and advancement of various types of cancer (Lee et al., 2009). The inflammasome is an intracellular complex composed of multiple proteins that activates the inflammatory response in tissues when exposed to various stimuli. Over 20 inflammasomes have been identified, among which the NLRP3 inflammasome has been extensively studied and widely recognized for its crucial involvement in inflammation (Moossavi et al., 2018). The NLRP3 inflammasome comprises three main components: the NOD-like receptor NLRP3, the adaptor protein apoptosis-associated speck-like protein containing a caspase recruitment domain (ASC), and pro-caspase-1. The activation of the NLRP3 inflammasome involves a two-step process. In the first step, pathogen-associated molecular patterns (PAMPs) or danger-associated molecular patterns (DAMPs) such as viruses, bacteria, or lipopolysaccharide (LPS) stimulate the expression of NLRP3, pro-IL-1β, and pro-IL-18. In the second step, a secondary stimulus like adenosine 5′-triphosphate (ATP), silica, or monosodium urate (MSU) triggers the activation of the NLRP3 inflammasome. This leads to the assembly of the NLRP3, ASC, and pro-caspase-1 components, resulting in the cleavage of pro-IL-1β and pro-IL-18 into their active forms, and the subsequent release of these pro-inflammatory cytokines (He et al., 2016). An increasing body of evidence indicates that the NLRP3 inflammasome plays a pivotal role in the development and progression of various types of cancer, including gastrointestinal cancer, skin cancer, breast cancer, hepatocellular carcinoma, and lung cancer (Huang et al., 2019; Gouravani et al., 2020; Lin et al., 2021; Xu et al., 2021). Activation of the NLRP3 inflammasome leads to the release of inflammatory cytokines including IL-1β and IL-18 to promote tumorigenesis (Gouravani et al., 2020). The activation of the inflammasome has also been implicated in promoting the migration and metastasis of various types of tumor cells (Dupaul-Chicoine et al., 2015; Yao et al., 2019). Given these findings, targeting the NLRP3 inflammasome holds potential as a therapeutic strategy for the treatment of NSCLC.

*Sophora exigua* Craib, commonly referred to as “Phit sa nat” in Thai, is a plant belonging to the Fabaceae family and is found in different regions of Thailand. This Thai traditional medicinal plant, *S. exigua*, is known for its antipyretic and anti-inflammatory properties (Krishna et al., 2012; Wang et al., 2016a). *S. exigua* is commonly incorporated into various multi-herb medicinal preparations, including the traditional Thai formula known as “Kheaw-Hom” remedy. This traditional remedy, Kheaw-Hom, is recognized and listed in the National List of Essential Medicines 2011 in Thailand. This remedy consists of eighteen Thai medicinal plants (Sukkasem, 2015). For an extended period, *S. exigua* has been utilized in folk medicine as a treatment for fever, skin ailments, measles, and chickenpox (Sukkasem et al., 2016). Several species within the Sophora genus have been studies for their pharmacological activities and potential therapeutic applications. Some of the most notable species include *Sophora flavescens*, *Sophora japonica* and *Sophora alopecuroides* (Krishna et al., 2012). These species have a long history of use in traditional medicine, particularly in East Asian countries (Aly et al., 2019; Abd-Alla et al., 2021). It is worth noting that specific medical research for *Sophira exigua* may be limited. *S*. *exigua* root extract exhibited antioxidant and antimalarial properties in plasmodium berghei-infected mice (Kaewdana et al., 2021). The root extract of *S. exigua* has demonstrated antibacterial activity against methicillin-resistant *Staphylococcus aureus* (MRSA), indicating its potential in combating this antibiotic-resistant bacterial strain (Tsuchiya et al., 1996; Fakhimi et al., 2006). In Folk medicine, it is used in the treatment of fever and inflammation (Sukkasem et al., 2016). However, the effect of *S*. *exigua* root extract and its chemical constituents on NLRP3 inflammasome pathway in NSCLC remains largely unknown. Therefore, the underlying mechanisms and potential therapeutic applications of *S. exigua* and its active compounds on the anti-inflammation-related cancer properties should be explored.

In this study, the *in vitro* anti-inflammation-related cancer properties of *S*. *exigua* root extract and its bioactive compounds on NSCLC cell lines were investigated. The A549 cells were primed with lipopolysaccharide (LPS) and adenosine triphosphate (ATP) for activated NLRP3 inflammasome and treatment with *S*. *exigua* root extract or its bioactive compounds. The study examined the anti-inflammatory, anti-proliferative, and anti-metastatic properties of *S. exigua*. The findings revealed that *S. exigua* root extract and its bioactive compounds were able to suppress the proliferation and metastasis of NSCLC cells by inhibiting the activation of the NLRP3 inflammasome. These results provide scientific evidence supporting the potential use of *S. exigua* root extract or its bioactive compounds as therapeutic strategies for NSCLC, specifically by targeting the NLRP3 inflammasome pathway.

## Materials and methods

### Chemical and reagents

Dulbecco’s Modified Eagle Medium (DMEM) medium, penicillin-streptomycin, and Fetal bovine serum (FBS) were obtained from Gibco BRL Company (Grand Island, NY, United States). Protease inhibitor cocktail, Commasie Plus™ Protein Assay Reagent, Modified Radioimmunoprecipitation assay (RIPA) lysis buffer, and chemiluminescent immunoblotting reagent were purchased from Thermo Fisher Scientific (Rockford, IL, United States). Primary antibodies for cyclin D1, cyclin E, CDK-2, CDK-4, anti-caspase-1 (p50 and p20), anti-NLRP3, anti-ASC, and horseradish peroxidase-conjugated anti-mouse or rabbit-IgG were purchased from Cell Signaling Technology (Beverly, MA, United States). Primary antibodies for β-actin and propidium iodide (PI) dye were obtained from Sigma-Aldrich (St. Louis, MO, United States). ReverTra Ace^®^ qPCR Master Mix was purchased from Toyobo Co., Ltd. (Osaka, Japan). SensiFAST SYBR Lo-ROX Kit was obtained from Meridian Bioscience^®^ (Cincinnati, OH, United States).

### Herb materials and preparation of *Sophora exigua* extracts

*Sophora exigua* was collected from natural source in 2022 from Chaiyaphum province, Thailand. Plant materials were identified and validated by Angkhana Inta, Chiang Mai University, Sukanda Chaiyong, Trinnakorn Katekulaphan, and Methee Rungrojsakul, Chandrakasem Rajabhat University, Bangkok, Thailand. The voucher specimen numbers of *S. exigua* (No. WP6605, WP6606, and WP7184) were certified by the herbarium at the Queen Sirikit Botanical Garden, Chiang Mai province, Thailand which was kept for future reference. The air-dried roots of *S. exigua* plants (100 g) were collected from 12 different sources and subjected to extraction using hexane (500 mL × 3 times), ethyl acetate (500 mL × 3 times), and ethanol (500 mL × 3 times). The resulting hexane, ethyl acetate, and ethanol extracts were filtered and concentrated under reduced pressure, yielding three crude fractional extracts.

### Column chromatography

In the experimental procedure, silica gel grade 60 was employed as the stationary phase, packed into a 3 cm × 60 cm column. The initial crude fractional extract, weighing 5.0 g, was carefully applied to the upper portion of the silica gel within the column. To achieve effective separation of compounds, we employed varying ratios of hexane and ethyl acetate as mobile phase components. This strategic adjustment of the hexane and ethyl acetate ratios, which enhances polarity, played a crucial role in facilitating the separation of compounds of interest. Upon elution, fractions were systematically collected in individual test tubes, with each fraction comprising 8 mL of the eluted material. For the analysis of compound separation and identification, thin layer chromatography (TLC) was employed. A small aliquot from each fraction was spotted onto a TLC plate. The TLC plate was then introduced into a chamber containing a hexane and ethyl acetate mixture in a 2:1 ratio. Concurrently, a reference standard, specifically the crude fractional extract’s ethyl acetate fraction, was also subjected to TLC analysis alongside the fractions. Once the solvent front had progressed approximately 0.5 cm from the top of the TLC plate, the plate was removed from the chamber and subjected to molybdate staining. In order to identify and isolate the desired sub-fraction containing active compounds, fractions exhibiting similar TLC patterns were meticulously combined. The verification of purity for the consolidated fractions was carried out using TLC. Subsequently, the prominent compounds present in the consolidated fractions underwent further analysis to elucidate their chemical structures. This was achieved through the utilization of nuclear magnetic resonance (NMR) spectroscopy, specifically employing a Bruker instrument (Bruker, Fällanden, Switzerland).

### Total phenolic content

The total phenolic content of the *S. exigua* extracts used in this study was determined using a Folin-Ciocalteu assay. The Folin-Ciocalteu assay was used as a gold standard protocol for determination of total phenolic content that was descripted in the previous studies (Box, 1983; Noreen et al., 2017; Aryal et al., 2019). In summary, 0.4 mL of each concentration of the *S. exigua* extracts was mixed with 0.3 mL of 10% Folin-Ciocalteau reagent and kept in the dark at room temperature for 3 min. Subsequently, 0.3 mL of carbonated sodium (Na_2_CO_3_) was added to the mixture and was further incubated in the dark at room temperature for an additional 30 min. The absorbance of the resulting mixture was measured at 765 nm using a UV-visible spectrophotometer (UV-1800, SHIMADZU CO., LTD). The absorbance values were then compared to a standard curve of gallic acid (GA). The total phenolic content of the *S. exigua* extracts was expressed as milligrams of gallic acid equivalents per Gram of the herbal extracts (mg GAE/g extract).

### Total flavonoid content

The total flavonoid content of the *S. exigua* extracts was determined using the aluminum chloride (AlCl_3_) colorimetric assay with minor modifications from a previous study (Pękal and Pyrzynska, 2014). Catechin was used as the gold standard for comparison. In this assay, each concentration of the *S. exigua* extracts (250 µL) was mixed with 5% NaNO_2_ (125 µL) and incubated for 5 min. Subsequently, 10% AlCl_3_ (125 µL) was added to the mixture, followed by an additional 5 min of incubation. Afterward, 1.0 mL of NaOH was added, and the mixture was incubated for 15 min at room temperature. The absorbance of the resulting mixture was measured at 510 nm using a spectrophotometer and compared to a standard catechin. The total flavonoid content was expressed as milligrams of catechin equivalents (CE) per Gram of extract (mg CE/g extract).

### Cell culture

The human lung adenocarcinoma cell line (A549) (CCL-185™) was obtained from American Type Culture Collection (ATCC) (Manassas, VA, United States). The cells were cultured in Dulbecco’s Modified Eagle Medium (DMEM) medium supplemented with 10% FBS, 50 IU/mL penicillin, and 50 μg/mL streptomycin. The cells were maintained in a humidified incubator at 37°C with an atmosphere consisting of 95% air and 5% CO_2_. When the cells reached 70%–80% confluency, they were harvested and seeded for subsequent experiments.

### The cell viability assay

The cytotoxicity of *S. exigua* extracts and its active compounds, exiguaflavanone A (EGF-A) and exiguaflavanone B (EGF-B), against A549 cells was determined by a sulforhodamine B (SRB) assay as was previously described (Vichai and Kirtikara, 2006; Sodde et al., 2015; Kabała-Dzik et al., 2017). In this study, we aimed to assess the cytotoxicity of *S. exigua* extracts and its active compounds over the course of our experiment, spanning 24 and 48 h. Thus, we made slight modifications to the protocols to tailor them to our specific experimental conditions. These modifications allow us to capture the dynamic effects of the test compounds within our desired timeframe, enhancing the precision of our investigation. Briefly, A549 cells (3 × 10^3^ cells/well) were seeded in a 96-well plate and incubated at 37°C, 5% CO_2_ overnight. After that, the cells were treated with or without various concentrations of *S. exigua* extracts (0–500 μg/mL) and EGF-A (0–118 μM; MW = 424.5 g/mol) and EGF-B (0–114 μM; MW = 438.5 g/mol) for 24, and 48 h to observe the dose-response curve. Following incubation, the cells were treated with 10% (w/v) trichloroacetic acid (TCA) and incubated at 4°C for 1 h. Subsequently, the medium was aspirated, and the cells were rinsed with gently running tap water. Next, 100 μL of a 0.054% (w/v) solution of sulforhodamine B (SRB) was added to each well, and the cells were incubated at room temperature for 30 min. After the incubation period, the SRB solution was removed, and the cells were washed four times with 1% (v/v) acetic acid. The cells were then allowed to dry at room temperature. To dissolve the dye, 150 μL of a 10 mM tris-based solution (pH 10.5) was added to each well, and the absorbance was measured at 510 nm using a microplate reader. The cell viability was calculated by comparing the absorbance of the treated cells to that of the control cells.

### Determination of cytokine production

The levels of pro-inflammatory cytokines, specifically IL-1β, IL-18, and IL-6, in the cell culture supernatants were measured using an ELISA kit sourced from Biolegend (San Diego, CA, United States), following the manufacturer’s protocol as previously outlined (Arjsri et al., 2022). A549 cells were seeded in a 6-well plate at a density of 2 × 10^5^ cells per well. After overnight incubation, the cells were treated with various concentrations (0–7.5 μg/mL) of *S. exigua* extracts or its active compounds, exiguaflavanone A (EGF-A) and exiguaflavanone B (EGF-B) (0–4 μg/mL), for 4 h. Lipopolysaccharide (LPS) and adenosine triphosphate (ATP) could stimulate the inflammatory responds, one of them was NLRP3 inflammasome pathway (Wang et al., 2016b; Huang et al., 2017). Therefore, the cells were induced by LPS at a concentration of 1,000 ng/mL for 18 h, followed by the ATP at a concentration of 5 nM for an additional 6 h. The culture supernatant was collected for ELISA analysis to measure cytokine release. Calibration standards for IL-6, IL-1β, and IL-18 (ranging from 0 to 500 pg/mL) were utilized to establish a standard curve for the ELISA assay as an internal quality control during the quantitative ELISA. The amount of cytokine released into the supernatant was calculated and compared to the standard curves provided by the ELISA kit.

### Colony formation assay

The inhibitory effect of *S. exigua* extract and its active compounds, exiguaflavanone A (EGF-A) and exiguaflavanone B (EGF-B), on A549 cells proliferation were determined using a colony formation assay, as per the established methodology outlined in the previous protocol (Franken et al., 2006). The A549 cells were seeded at 500 cells/well in a 6-well plate and incubated for 24 h. The cells were pre-treated with 0–40 μg/mL of SE-EA and 0–15 μg/mL of EGF-A and EGF-B for 24 h. Then, the cells were induced by lipopolysaccharide (LPS) at concentration 1,000 ng/mL for 18 h following with adenosine triphosphate (ATP) 5 nM for further 7 days before harvest. The cells were fixed with 6% glutaraldehyde for 30 min and stained with Toluidine dye for 15 min. The dye was removed by water. The stained cells were visualized, and the images were captured using the iBright™ CL-1500 imaging system (Thermo Fisher Scientific). The colony numbers were calculated using the “Analyze Particles.” function in ImageJ 1.410 software (https://imagej.nih.gov/ij/). The parameters were set to include colonies of provide specific parameters used, e.g., size range, circularity, etc., ensuring accurate quantification. In each experiment, determinations were carried out in triplicate.

### Cell cycle assay

To investigate the distribution of the cell cycle, we followed a previously established method (Crowley et al., 2016). A549 cells were seeded at a density of 1 × 10^5^ cells per well in a 6-well plate and cultured with 0.5% fetal bovine serum (FBS) in DMEM for 18 h. After the starvation period, the cells were treated with various doses of SE-EA or its active compounds, exiguaflavanone A (EGF-A), and exiguaflavanone B (EGF-B). The cells were incubated with SE-EA or EGF-A or EGF-B for 4 h. Then, the cells were induced by lipopolysaccharide (LPS) at concentration 1,000 ng/mL for 18 h following with adenosine triphosphate (ATP) 5 nM for further 6 h before harvest. Following the treatment, the cells were harvested and processed for cell cycle analysis. This typically involves trypsinizing the cells to detach them from the culture plate, fixing them in ice-cold 70% ethanol in −20°C overnight. After washing the cell pellets with PBS, 12.5 µL of ribonuclease A (RNase A) was added to the pellets and incubated at 37°C in a CO_2_ incubator for 15 min. Following the RNase A treatment, 200 µL of propidium iodide (PI) dye was added to the cell pellets and incubated at 37 °C in a CO2 incubator for 45 min. After the incubation period, the cells were centrifuged to remove the PI dye and the cell pellets were then re-suspended with 500 µL of PBS. The stained cells would then be subjected to flow cytometry analysis (Beckman Coulter Inc., Indiana, United States) to determine the distribution of cells in different phases of the cell cycle (G0/G1, S, and G2/M).

### Wound-scratch assay

The wound-scratch assay was conducted to examine the migratory capacity of A549 cells as per the established protocol (Liang et al., 2007). Briefly, A549 cells were seeded at 2.5 × 10^5^ cells in a 6-well plate and incubated for 24 h and then were cultured with 0.5% FBS in DMEM for overnight. A549 cells were cultured to 90%–100% confluence, a 200 µL pipette tip was used to generate a wound in the surface of the cells in a 6-well plate, and then the cells were treated with 0–7.5 μg/mL of SE-EA or (0–4 μg/mL) of its active compounds, exiguaflavanone A (EGF-A) and exiguaflavanone B (EGF-B) for 4 h. Then, the cells were induced by lipopolysaccharide (LPS) at concentration 1,000 ng/mL for 18 h following with (ATP) adenosine triphosphate 5 nM for further 6 h. Images of the same fields at the indicated time-points (0 and 24 h) were captured under a light microscope at a magnification of ×100. The images were captured under a phase-contrast microscope (Nikon Eclipse TS100, NIKON INSTRUMENT INC). The wound area was calculated using the " Wound healing size” plugin in ImageJ 1.410 software (Suarez-Arnedo et al., 2020). The plugin parameters were adjusted to accurately delineate the wound area for each experimental condition.

### Trans-well migration and invasion assay

To evaluate the impact of SE-EA (*S. exigua* extract), exiguaflavanone A (EGF-A), and exiguaflavanone B (EGF-B) on the invasion and migration of A549 cells, trans-well migration and invasion assays were performed following the previously outlined protocol (Pitchakarn et al., 2012). Polyvinylpyrrolidone-free polycarbonate filters with 8 μm pore size (BD Biosciences, Franklin Lakes, NJ, United States) were used. For the invasion assay, the filters were coated with 15 μg of Matrigel per filter, while for the migration assay, the filters were coated with 10 μg/mL fibronectin. A549 cells were seeded into the upper chamber of the trans-well inserts at a density of 5 × 10^4^ cells per well. The cells were cultured in 0.1% FBS DMEM containing different concentrations of SE-EA, EGF-A, or EGF-B (0–7.5 μg/mL for SE-EA and 0–4 μg/mL for EGF-A and EGF-B). The lower chamber of the trans-well was filled with 10% FBS DMEM, which acts as a chemoattractant. After 4 h of incubation, the cells were induced by lipopolysaccharide (LPS) at a concentration of 1,000 ng/mL for 18 h, followed by the addition of adenosine triphosphate (ATP) at a concentration of 5 nM for an additional 6 h. The invading or migrating cells on the lower surface of the trans-well filter were fixed with 95% ethanol for 5 min and then stained with 0.5% crystal violet in 20% methanol for 30 min. The stained cells were visualized under a phase-contrast microscope (Nikon Eclipse TS100, NIKON INSTRUMENT INC.), and images were captured. To quantify the extent of invasion or migration, the percentages of the areas occupied by cells were determined using the “Threshold” function in ImageJ 1.410 software (https://imagej.nih.gov/ij/). The threshold parameters were fine-tuned to distinguish cells and background, ensuring reliable assessment.

### Determination of IL-6, IL-1β, IL-18 and NLRP3 gene expressions by RT-qPCR analysis

To quantify the gene expressions of IL-6, IL-1β, IL-18 and NLRP3 at 24 h after LPS + ATP induction, we adopted the RT-qPCR analysis method described by (Olcum et al., 2021; Semmarath et al., 2023). A549 cells were pre-treated with 0–7.5 μg/mL of *S. exigua* extracts or (0–4 μg/mL) of its active compounds, exiguaflavanone A (EGF-A) and exiguaflavanone B (EGF-B) for 4 h. Then, the cells were induced by lipopolysaccharide (LPS) at concentration 1,000 ng/mL for 18 h following with (ATP) adenosine triphosphate 5 nM for further 6 h. After incubation, total mRNA was isolated from the cells using TRI reagent^®^, a commonly used reagent for RNA extraction. The concentration and purity of the isolated RNA were determined using NanoDrop™ 2000/2000c Spectrophotometers (Thermo Fisher Scientific, Waltham, MA, United States of America), which measure the absorbance of the RNA sample at specific wavelengths. The A260/A280 ratio (>1.8) indicates pure RNA without contamination. To obtain cDNA (complementary DNA), reverse transcription was performed using a Mastercycler^®^ nexus gradient machine (Eppendorf, GA, Germany). For quantitative real-time PCR (qRT-PCR), the cDNA samples were amplified and quantified using a qRT-PCR ABITM 7500 Fast and 7500 real-time PCR machine (Thermo Fisher Scientific, Waltham, MA, United States). This technique allows for the detection and quantification of specific target genes in the samples. According to the internal quality control during the qPCR, deionized water was use as a no-template control in every experiment. To assess the precision of our assays and ensure the reliability of the data, the duplicate runs have been performed for each sample and control. The gene expressions were analyzed using the software provided with the QuantStudio6 Flex real-time PCR system (Applied Biosystems). The 2^−ΔΔCT^ method with normalization to GAPDH and controls was used for calculation of results. All primer sequences used in this study were as follows: IL**-**6 forward, 5**′-**ATG AAC TCC TTC ACA AGC**-**3**′**, reverse, 5**′-**GTT TTC TGC CAG TGC CTC TTT G**-**3**’**; IL-1β forward, 5**′-** ATG ATG GCT TAT TAC AGT GGC AA**-**3**′**, reverse, 5**′-** GTC GGA GAT TCG TAG CTG GA **-**3**′**; IL-18 forward, 5**′-** AAA CTA TTT GTC GCA GGA ATA AAG AT **-**3**′** reverse, 5**′-** GCT TGC CAA AGT AAT CTG ATT CC **-**3**′**; NLRP3 forward, 5**′-** AAG GGC CAT GGA CTA TTT CC **-**3**′** reverse, 5**′-** GAC TCC ACC CGA TGA CAG TT **-** 3**′**and GAPDH forward, 5**′-** TCA ACA GCG ACA CCC AC **-**3**′** reverse, 5**′-** GGG TCT CTC TCT TCC TCT TGT G **-**3**′ (**Humanizing Genomics Macrogen, Geumcheon-gu, Seoul, South Korea**)**.

### Western blot analysis

To determine the expression of cell cycle regulator proteins (CDK-2, CDK-4, cyclin D1 and cyclin E), The cells (5 × 10^5^ cells per well) were plated in 6-well plate and cultured with 0.5% FBS in DMEM for 18 h before they were treated with 0–7.5 μg/mL of SE-EA or 0–4 μg/mL of its active compounds, exiguaflavanone A (EGF-A) and exiguaflavanone B (EGF-B) for 4 h. Then, the cells were induced inflammation by LPS at concentration 1,000 ng/mL for 18 h followed by ATP adenosine triphosphate 5 nM for further 6 h before harvested. In order to determine the effects of SE-EA or its active compounds, exiguaflavanone A (EGF-A) and exiguaflavanone B (EGF-B) on NLRP3 inflammasome pathway, the A549 cells were pre-treated with 0–7.5 μg/mL of SE-EA or 0–4 μg/mL of its active compounds, exiguaflavanone A (EGF-A) and exiguaflavanone B (EGF-B) for 4 h. After inducing inflammation by lipopolysaccharide (LPS) and adenosine triphosphate (ATP) for 6 h, the cells were harvested, and the resulting cell pellets were lysed using RIPA buffer, adhering to the methodology outlined in the previous study (Chittasupho et al., 2023). RIPA buffer contains detergents and salts that help to solubilize and extract proteins from the cells. The protein concentration in the lysate was determined using the Bradford assay, which is a commonly used method for protein quantification. The Bradford assay relies on the binding of Coomassie Brilliant Blue dye to proteins, resulting in a color change that can be measured spectrophotometrically. For protein analysis, the whole-cell lysate was subjected to 10% SDS-PAGE (sodium dodecyl sulfate-polyacrylamide gel electrophoresis). To prevent non-specific binding, the membranes were blocked with 5% bovine serum albumin (BSA) in 0.5% TBS-Tween. After blocking, the membranes were washed with 0.5% TBS-Tween to remove excess BSA and unbound proteins. Following the cyclin D1, cyclin E1, CDK-2, CDK-4, NLRP3, ASC, or caspase-1 primary antibody incubation, the membranes were washed multiple times with 0.5% TBS-Tween to remove any unbound primary antibody. Subsequently, the membranes were incubated with a horseradish peroxidase (HRP)-conjugated secondary antibody, either anti-mouse or anti-rabbit IgG, depending on the primary antibody used. Finally, the membranes were exposed to an imaging system such as the iBright™ CL-1500 imaging system (Thermo Fisher Scientific) to capture the chemiluminescent signal. The resulting images were used for the analysis of band density levels using the “Measure” function in ImageJ 1.410 software (https://imagej.nih.gov/ij/).

### Statistical analysis

The experiments were performed in triplicate independent experiments to ensure reproducibility. The data obtained from each experiment were presented as the mean ± standard deviation (mean ± SD), which provides an indication of the variability within the data set. Statistical analysis was performed using Prism version 8.0 software, which is a commonly used statistical analysis tool. The independent *t*-test and one-way ANOVA with Dunnett’s test were used for data analysis in various experiments. The significance level for determining statistical significance was set at *p < 0.05, **p < 0.01, and ***p < 0.001. These values indicate the probability of obtaining the observed results by chance. A smaller *p*-value indicates a higher level of statistical significance.

## Results

### Extraction of *Sophora exigua* and phytochemicals study

**FIGURE 1:**
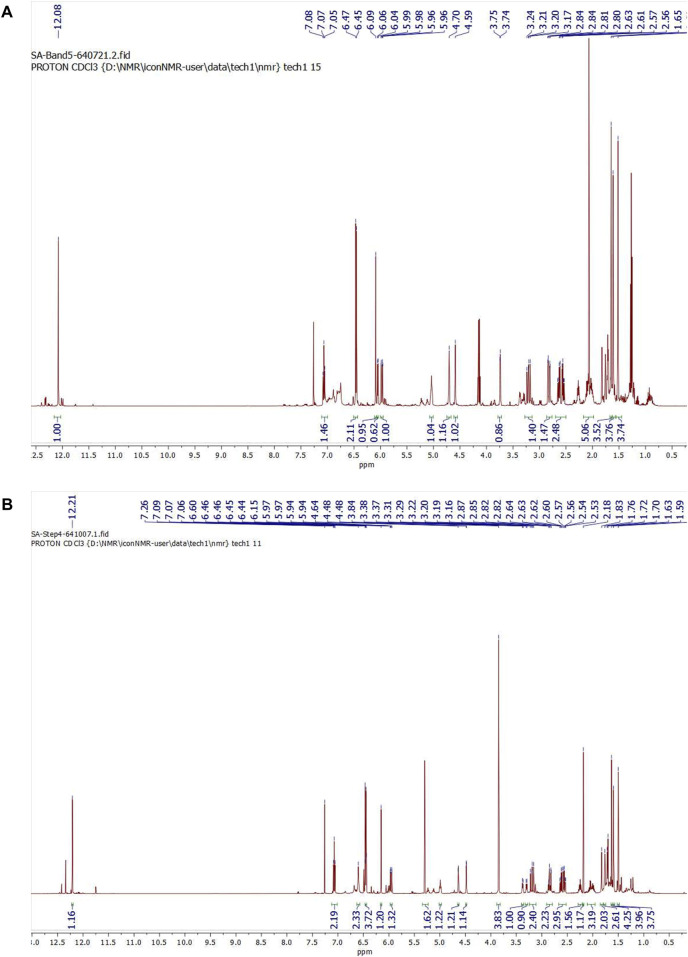
^1^H NMR spectrums of purified compound spot **(A)** No. 1 and **(B)** No. 2 which were identified to be as exiguaflavanone A and exiguaflavanone B, respectively.

After subjecting *S. exigua* to solvent partition extraction, three fractions were obtained: *S. exigua* hexane extract (SE-Hex), *S. exigua* ethyl acetate extract (SE-EA), *S. exigua* ethanolic extract (SE-EtOH). Table 1 displays the total phenolic and total flavonoid contents of each fraction. The results indicate that SE-EA has the highest total phenolic content (152.12 ± 8.73 mg of GAE/g extract) and the total flavonoid content (112.75 ± 4.32 mg of CE/g extract), followed by SE-EtOH and SE-Hex respectively. Thus, the solvent partition extraction technique can increase the phenolic content in the extracts, as observed in SE-EA, this fraction can be used further to identify the bioactive phytochemical compounds present. Among the three fractional extracts tested, the ethyl acetate extract had the highest yield, with a value of 15.8 ± 0.05, followed by the ethanolic and hexane extracts with values of 4.53 ± 0.06, and 3.88% ± 0.09%, respectively. The ethyl acetate extract (5 g) was further purified using silica gel column chromatography, yielding two dominant compounds with yields of 3.94% and 5.96%, respectively. The compounds were identified as exiguaflavanone A and exiguaflavanone B through comparison of their ^1^H-NMR spectra (Figure 1; Tables 2, 3) with previously published data (Ruangrungsi et al., 1992). Exiguaflavanone A was obtained as a brown gum-like material (purity degree is >90%), while exiguaflavanone B was obtained as a yellow viscous oil (purity degree is >90%).

**TABLE 1:** Phytochemical study of *S*. *exigua* extracts.

*Sophora exigua* extracts	Total phenolic content (mg GAE/g extract)	Total flavonoid content (mg CE/g extract)
SE-Hex	18.57 ± 3.27	10.59 ± 1.58
SE-EA	152.12 ± 8.73***	99.29 ± 11.17***
SE-EtOH	39.78 ± 1.40	7.92 ± 1.94

^***^ *p* < 0.001 vs. others *S*. *exigua* extracts using independent *t*-test. Data are presented as mean ± S.D., values of three independent experiments.

**TABLE 2:** ^1^H-NMR spectral data of Exiguaflavanone A in comparison to those previously reported by Ruangrungsi et al. (Ruangrungsi et al., 1992).

Position	Exiguaflavanone A in CDCl_3_ (our results, 500 MHz)	Exiguaflavanone A in acetone-d_6_ (Ruangrungsi’s results)
	*δ*_H_ (*multi*., *J* (Hz), integral)	*δ*_H_ (*multi*., *J* (Hz), integral)
2	5.97 (*dd*, 13.8, 2.8, 1H)	6.02 (*dd*, 14, 3, 1H)
3	3.21 (*dd*, 17.6, 13.8, 1H)	3.87 (*dd*, 17, 14, 1H)
	2.82 (*dd*, 17.6, 2.8, 1H)	2.54 (*dd*, 17, 3, 1H)
5-OH	12.08 (*s*, 1H)	12.27 (*s*, 1H)
6	6.09 (*s*, 1H)	6.01(*s*, 1H)
7-OH	—	9.45 (*br s*, 1H)
2′, 6′-OH	—	8.50 (*br s*, 2H)
3′,5′	6.46 (*d*, 8.2, 2H)	6.47 (*d*, 8, 2H)
4′	7.07 (*t*, 8.1, 1H)	7.05 (*t*, 8, 1H)
1"	2.63 (*dd*, 13.9, 8.2, 1H)	2.56-2.60 (*m*, 3H)
	2.55 (*dd*, 14.0, 5.8, 1H)	
2"	2.27 (*m*, 1H)	
3"	2.01 (*m*, 2H)	2.08 (*m*, 2H)
4"	5.04 (*t*, 6.3, 1H)	4.98 (*t like*, 1H)
6"	1.61 (*s*, 3H)	1.54 (*br s*, 3H)
7"	1.52 (*s*, 3H)	1.48 (*br s*, 3H)
9"	4.70 (*br s*, 1H)	4.55 (*br s*, 2H)
	4.59 (*br s*, 1H)	
10"	1.65 (*s*, 3H)	1.61(*br s*, 3H)

**TABLE 3:** ^1^H-NMR spectral data of Exiguaflavanone B in comparison to those previously reported by Ruangrungsi et al. (1992).

Position	Exiguaflavanone B in CDCl_3_ (our results, 500 MHz)	Exiguaflavanone B in acetone-d_6_ (Ruangrungsi’s results)
	*δ*_H_ (*multi*., *J* (Hz), integral)	*δ*_H_ (*multi*., *J* (Hz), integral)
2	5.96 (*dd*, 13.7, 2.8, 1H)	6.02 (*dd*, 14, 3, 1H)
3	3.19 (*dd*, 17.6, 13.7, 1H)	3.89 (*dd*, 16, 14, 1H)
	2.84 (*dd*, 17.6, 2.8, 1H)	2.56 (*dd*,16, 3, 1H)
5-OH	12.20 (*s*, 1H)	12.38 (*s*, 1H)
6	6.15 (*s*, 1H)	6.13 (*s*, 1H)
7-OCH_3_	3.84 (*s*, 3H)	3.87 (*s*, 3H)
2′, 6′-OH	6.78 (*s*, 2H)	8.55 (*br s*, 2H)
3′,5′	6.46 (*d*, 8.2, 2H)	6.48 (*d*, 8, 2H)
4′	7.07 (*t*, 8.1, 1H)	7.03 (*t*, 8, 1H)
1"	2.62 (*dd*, 13.4, 8.2, 1H)	2.00-2.55 (*m*, 5H)
	2.55 (*dd*, 13.4, 6.0, 1H)	
2"	2.29-2.22 (*m*, 1H)	
3"	2.09-1.95 (*m*, 2H)	
4"	4.99 (*t*, 6.7, 1H)	4.94 (*t like*, 1H)
6"	1.59 (*s*, 3H)	1.53 (*br s*, 3H)
7"	1.50 (*s*, 3H)	1.47 (*br s*, 3H)
9"	4.64 (*m*, 1H)	4.45 (*br s*, 1H)
	4.48 (*d*, 2.1, 1H)	4.49 (*br s*, 1H)
10"	1.63 (*s*, 3H)	1.60 (*br s*, 3H)

### Effects of *S. exigua* extracts and its active compounds on A549 cells viability

**FIGURE 2:**
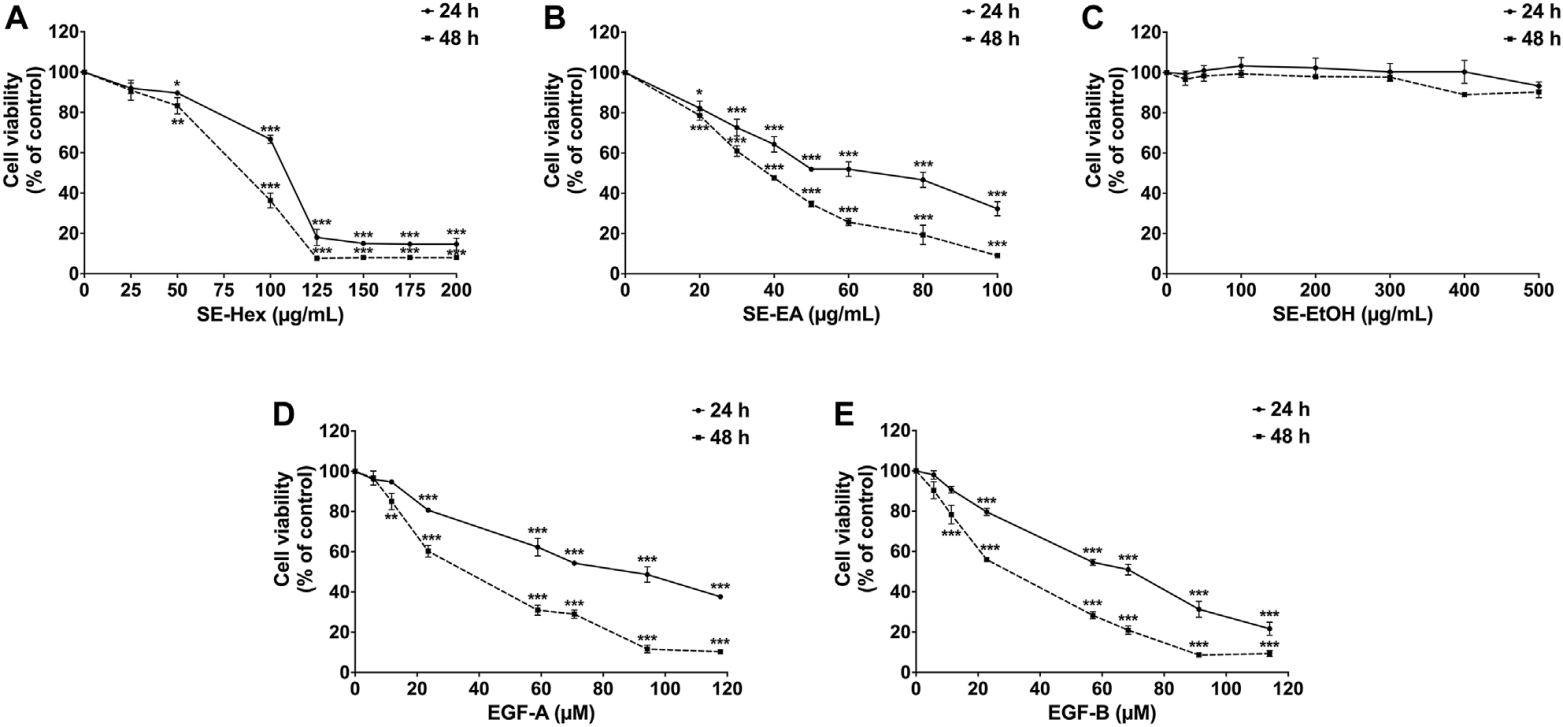
The effects of *S. exigua* extracts and its active compounds (EGF-A and EGF-B) on A549 cells viability were determined by SRB assay. The A549 cells were treated with 0–200 μg/mL of SE-Hex **(A)**, 0–100 μg/mL of SE-EA **(B)**, and 0–500 μg/mL of SE-EtOH **(C)** for 24, and 48 h. A549 cells were treated with 0–118 µM of EGF-A **(D)** and 0–114 µM of EGF-B **(E)** for 24 and 48 h. Data are presented as mean ± S.D. values of three independent experiments. *p < 0.05, **p < 0.01 and ***p < 0.001 compared to the control (0 μg/mL or 0 µM).

Prior to investigating the anti-inflammatory properties of *S*. *exigua* extracts and its bioactive compounds, the cytotoxic effects of SE-EA and its bioactive compounds on A549 cells were evaluated. The viability of A549 lung cells was measured using SRB assay after treatment with different concentrations of SE-Hex, SE-EA, and SE-EtOH, as well as exiguaflavanone A and exiguaflavanone B, for 24 and 48 h. The results revealed that SE-EA exhibited a cytotoxic effect on A549 cells, with an IC_50_ of 64.00 ± 6.46 and 37.00 ± 1.53 μg/mL after 24 and 48 h of incubation, respectively. On the other hand, SE-Hex had a higher IC_50_ value of 111.67 ± 7.64 and 85.00 ± 5.00 μg/mL after 24 and 48 h of incubation, respectively (Figure 2), indicating less cytotoxicity than SE-EA. SE-EtOH did not exhibit any cytotoxic effects on A549 cells at any concentration or time point tested. Exiguaflavanone A and exiguaflavanone B also showed cytotoxicity on A549 cells, with an IC_50_ of 88.74 ± 11.14 and 68.42 ± 7.48 µM, respectively after 24 h of incubation, and with an IC_50_ of 34.94 ± 6.05 and 30.03 ± 2.37 µM, respectively, after 48 h of incubation. Non-toxic concentrations of SE-Hex, SE-EtOH, SE-EA (0–7.5 μg/mL), and the active compounds exiguaflavanone A (0–9.42 µM or 0–4 μg/mL) and exiguaflavanone B (0–9.12 µM or 0–4 μg/mL) were selected for further experiments to investigate their anti-inflammatory properties on A549 cells.

### Anti-inflammatory effects of *S. exigua* extracts and its active compounds on LPS-ATP-induced A549 cells

**FIGURE 3:**
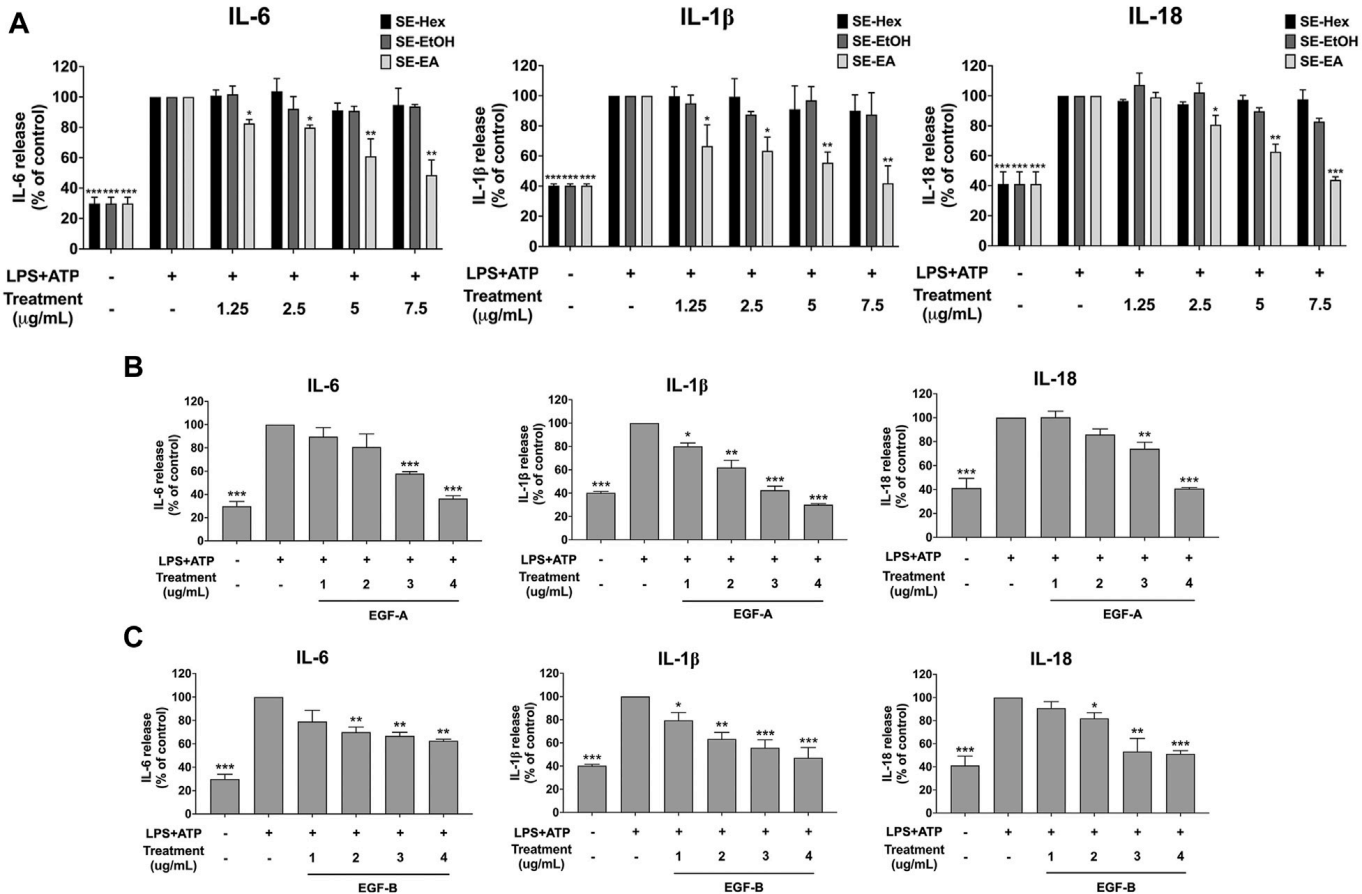
Inhibitory effects of *S. exigua* extracts and its active compounds (EGF-A and EGF-B) on the pro-inflammatory cytokine release in LPS-ATP-induced A549 cells. A549 cells were treated with *S. exigua* extracts **(A)**, SE-Hex, SE-EtOH, and SE-EA at a concentration of 0–7.5 μg/mL or active compounds, EGF-A **(B)** at a concentration of 0–4 μg/mL (0–9.42 µM) or EGF-B **(C)** at a concentration of 0–4 μg/mL (0–9.12 µM), for 4 h. Then, the cells were induced by lipopolysaccharide (LPS) at concentration 1,000 ng/mL for 18 h following with (ATP) adenosine triphosphate 5 nM for further 6 h. The IL-6, IL-1β and IL-18 releases into the culture supernatant were examined by ELISA. The LPS-ATP-induced A549 as 100%. Data are presented as mean ± S.D. values of three independent experiments, *p < 0.05, **p < 0.01 and ***p < 0.001 compared to the LPS-ATP-induced control group.

To assess the anti-inflammatory effects of *S*. *exigua* extracts (SE-Hex, SE-EtOH and SE-EA) and their bioactive compounds (exiguaflavanone A and exiguaflavanone B) on LPS-ATP-induced inflammation, we measured the release of pro-inflammatory cytokines (IL-6, IL-1β, and IL-18) in the supernatant of A549 cells using ELISA. The levels of these cytokines were significantly increased in LPS-ATP-induced A549 cells compared to non-induced cells (*p* < 0.001), as shown in Figure 3. Treatment with SE-EA resulted in a dose dependent decrease in the release of IL-6, IL-1β, and IL-18 from LPS-ATP-induced A549 cells (*p* < 0.001). However, SE-Hex and SE-EtOH did not exhibit any inhibitory effects on cytokine releases (IL-6, IL-1β, and IL-18), as shown in Figure 3A. Moreover, the bioactive compounds exiguaflavanone A and exiguaflavanone B also demonstrated significant inhibition of IL-6, IL-1β, and IL-18 release from LPS-ATP-induced A549 cells in a dose-dependent manner (*p* < 0.001), as shown in Figures 3B, C. These results indicate that SE-EA and its bioactive compounds, exiguaflavanone A and exiguaflavanone B, possess anti-inflammatory properties against LPS-ATP inducted inflammation by reducing the release of IL-6, IL-1β, and IL-18 cytokines in A549 cell culture supernatant.

### Effects of SE-EA and its active compounds (EGF-A and EGF-B) on LPS-ATP-induced A549 cells proliferation

**FIGURE 4:**
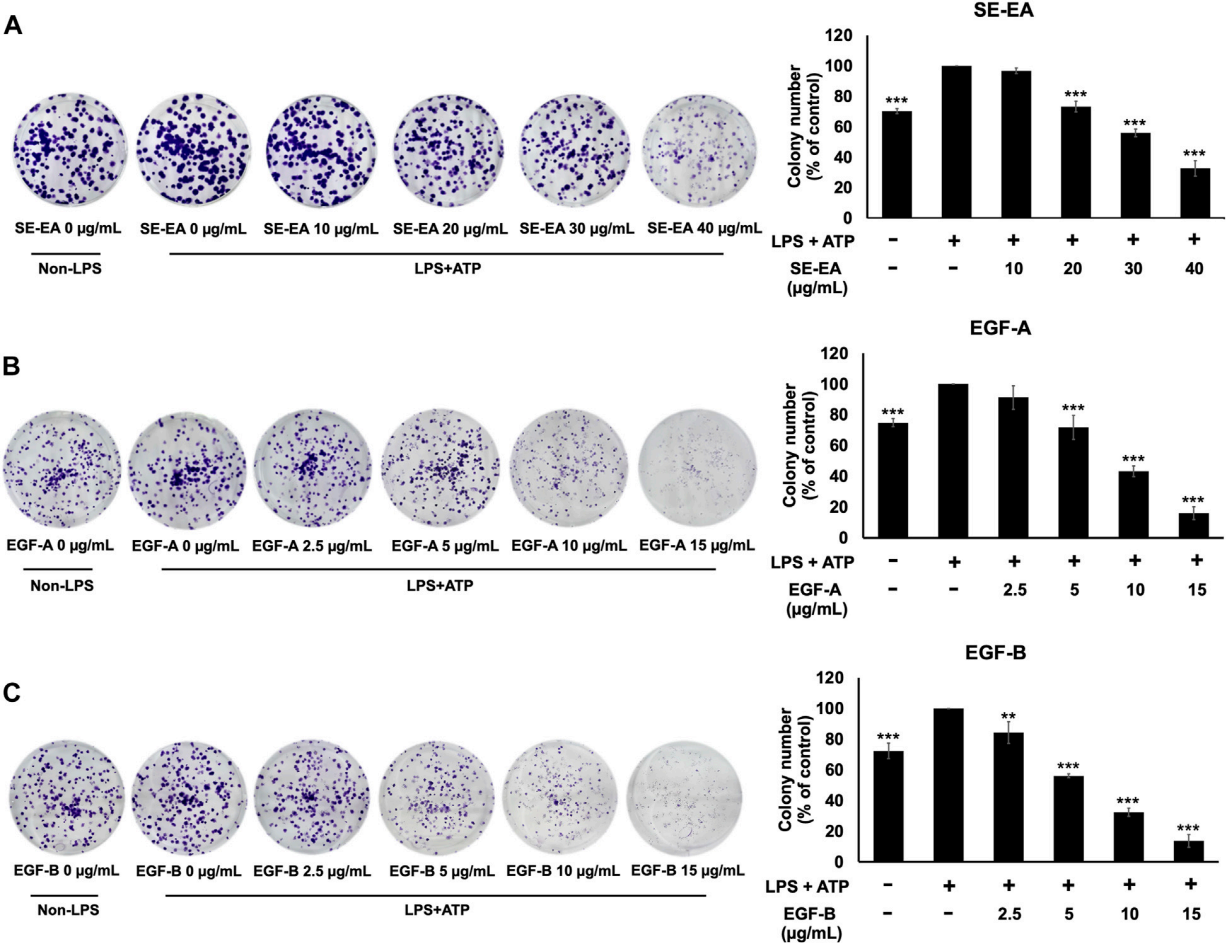
The effects of SE-EA and its active compounds (EGF-A and EGF-B) on LPS-ATP-induced A549 cells proliferation using a colony formation assay. A549 cells were treated with SE-EA **(A)** at 0–40 μg/mL, EGF-A **(B)** at 0–15 μg/mL (0–35.33 µM), and EGF-B **(C)** at 0–15 μg/mL (0–34.20 µM) for 7 days. The resulting colonies were photographed and quantified using ImageJ software. Data are presented as the mean ± S.D. of three independent experiments. *p < 0.05, **p < 0.01 and ***p < 0.001 compared to the LPS-ATP-induced control group.

**FIGURE 5:**
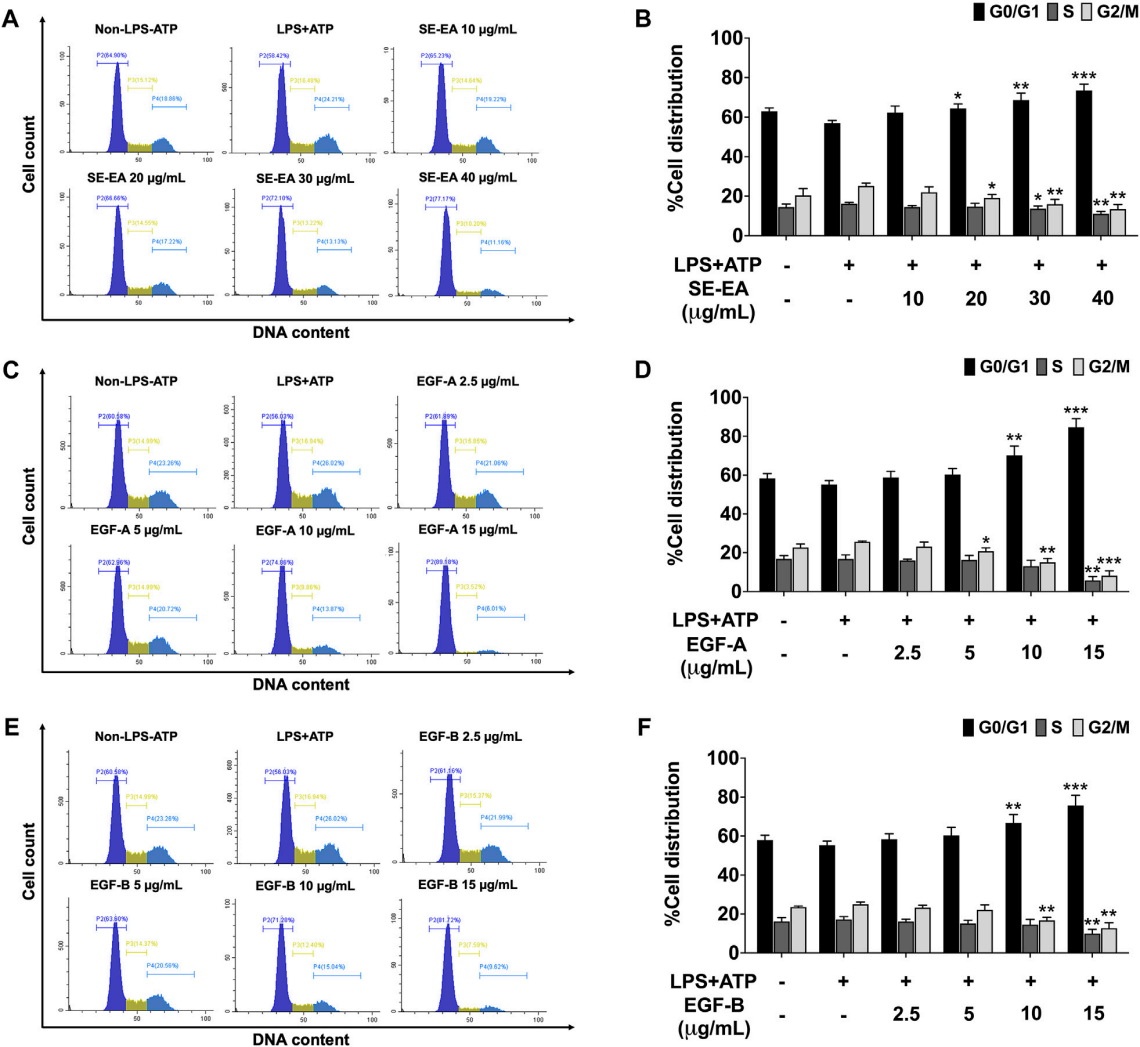
The effect of SE-EA and its active compounds (EGF-A and EGF-B) on cell cycle distribution in LPS-ATP-induced A549 cells. The cells were treated with SE-EA **(A, B)** at a concentration range of 0–40 μg/mL or active compounds, EGF-A **(C, D)** or EGF-B **(E, F)**, at a concentration range of 0–15 μg/mL (0–35.33 µM) and 0–15 μg/mL (0–34.20 µM), respectively, for 4 h. The cells were then induced by lipopolysaccharide (LPS) at a concentration 1,000 ng/mL for 18 h, followed by adenosine triphosphate (ATP) at 5 nM for a further 6 h. After staining the cells with PI dye, cell cycle distribution was analyzed by flow cytometry **(A, C, E)** and the percentages of cells in the G0, S and G2/M phase were quantified **(B, D, F)**. The data were obtained from three independent experiments and are presented as mean ± S.D. values. Statistical significance was determined with respect to the LPS-ATP-induced control group, and indicated as *p < 0.05, **p < 0.01 and ***p < 0.001.

**FIGURE 6:**
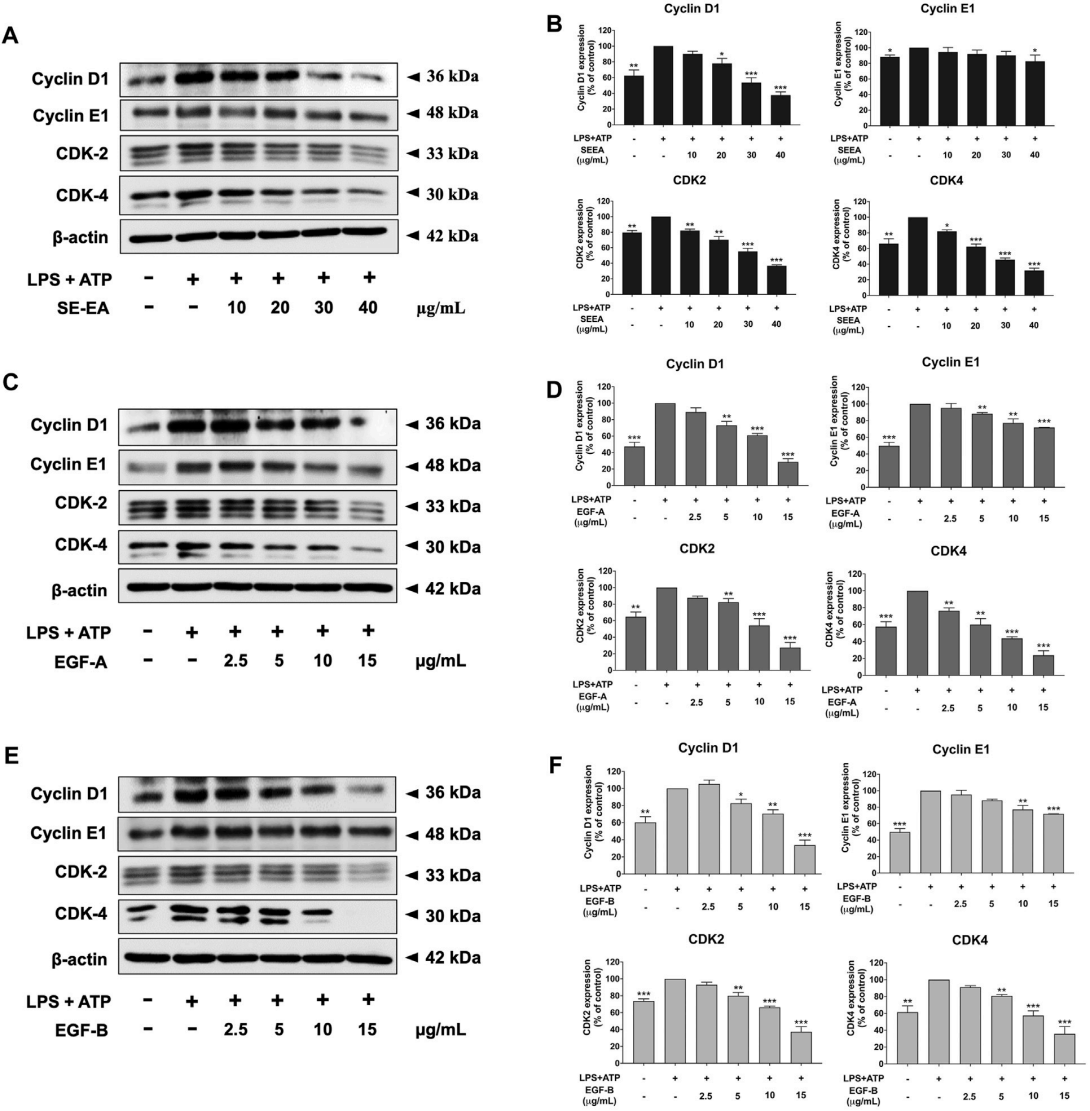
The effects of SE-EA and its active compounds (EGF-A and EGF-B) on cell cycle regulator proteins (Cyclin D1, Cyclin E1, CDK-2, and CDK-4) expression in LPS-ATP-induced A549 cells. The cells were treated with SE-EA **(A, B)** at concentrations of 0–40 μg/mL or active compounds, EGF-A **(C, D)** or EGF-B **(E, F)**, at a concentration range of 0–15 μg/mL (0–35.33 µM) and 0–15 μg/mL (0–34.20 µM), respectively, for 4 h. Following treatment, the cells were induced by lipopolysaccharide (LPS) at a concentration of 1,000 ng/mL for 18 h, followed by adenosine triphosphate (ATP) at a concentration of 5 nM for an additional 6 h before being harvested. The protein expression of Cyclin D1, Cyclin E1, CDK-2, and CDK-4 was determined by Western blot analysis, and the band density was measured using ImageJ 1.410 software. The LPS-ATP-induced A549 cells are presented as 100% of the control. Data are presented as mean ± S.D. values of three independent experiments. *p < 0.05, **p < 0.01 and ***p < 0.001 compared to the LPS-ATP-induced control group.

Based on previous research, it is known that the activation of NLRP3 inflammasome can promote cancer progression by releasing inflammatory cytokines such as IL-1β. In this study, A 549 lung cancer cells were induced with inflammation through the NLRP3 inflammasome pathways using LPS bacterial inflammation-inducing agents in combination with adenosine triphosphate (ATP), and measured cell proliferation using colony formation assay. The result indicated that lung cancer cells induced by LPS and ATP showed a greater ability to proliferate than lung cancer cells that were not induced with inflammation in A549 cells, as shown in Figure 4. Next, the effectiveness of SE-EA extract and its active compounds, EGF-A and EGF-B, in inhibiting the growth of A549 lung cancer cells were investigated, it was found that the SE-EA extract (Figure 4A) as well as EGF-A (Figure 4B) and EGF-B (Figure 4C) had significant inhibitory effects on the proliferation of A549 lung cancer cells induced by LPS and ATP. To further understand the mechanism by which SE-EA extract and its active compounds, EGF-A and EGF-B, inhibit cell growth, we conducted a cell cycle analysis assay. As shown in Figure 5, the results showed that the SE-EA (Figures 5A, B) and its active compounds EGF-A (Figures 5C, D) and EGF-B (Figures 5E, F) were able to arrest the growth of A 549 lung cancer cells induced by LPS and ATP at the G1 phase of the cell cycle. To gain insight into the underlying mechanism of SE-EA and its bioactive compounds, Western blotting analysis was conducted to assess the expression of cell cycle regulator proteins. The results showed that SE-EA, EGF-A, and EGF-B markedly reduced the expression of cyclin D1, cyclin E1, CDK-2, and CDK-4 in LPS-ATP-induced A549 cells in a dose-dependent manner, as shown in Figure 6.

### Effects of SE-EA and its active compounds (EGF-A and EGF-B) on LPS-ATP-induced A549 cells migration

**FIGURE 7:**
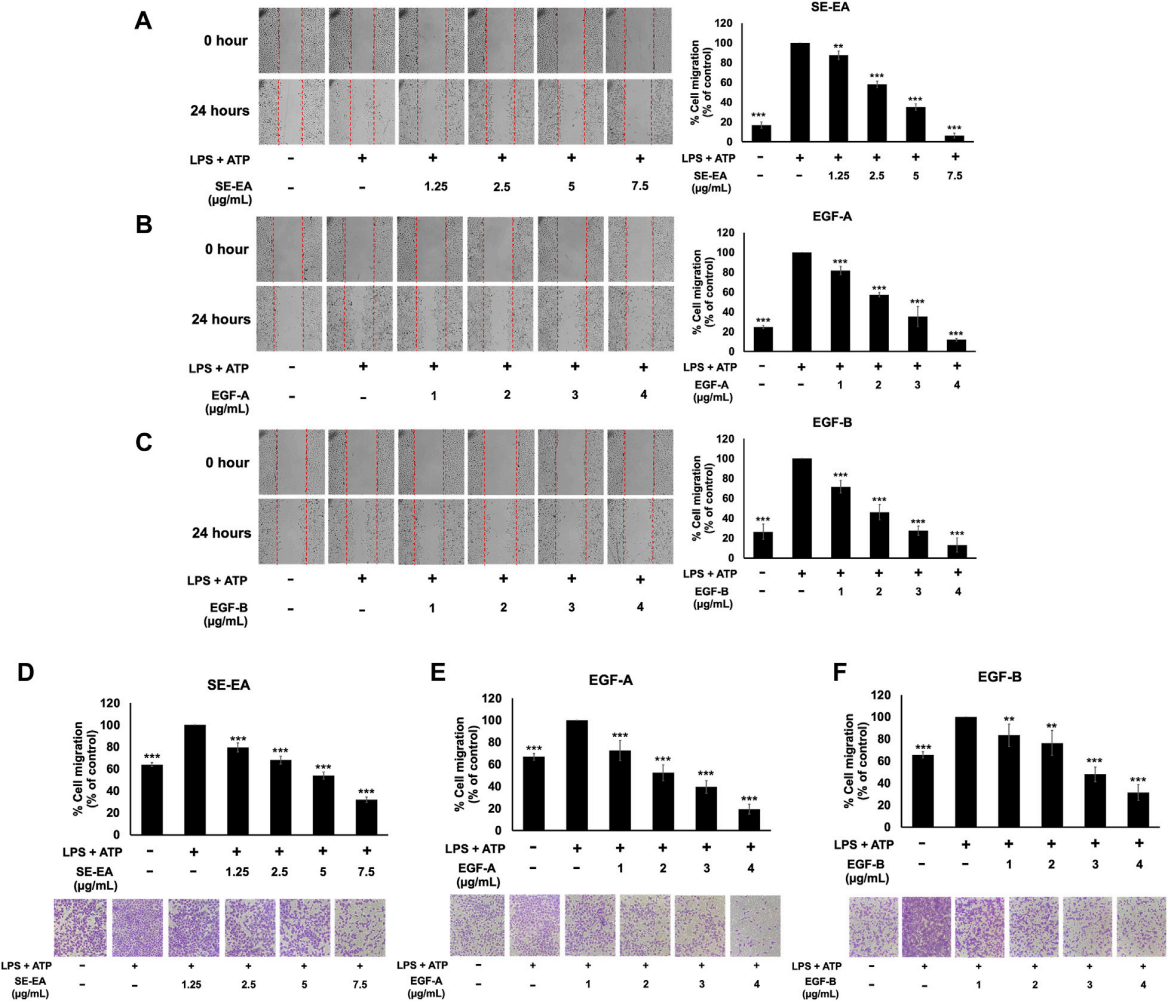
The effects of SE-EA and its active compounds (EGF-A and EGF-B) on the migration of LPS-ATP-induced A549 cells. A scratch assay was used to assess the anti-migration effects of SE-EA **(A)** at concentrations of 0–7.5 μg/mL, EGF-A **(B)** at concentrations of 0–4 μg/mL (0–9.42 µM), and EGF-B **(C)** at concentrations of 0–4 μg/mL (0–9.12 µM). Photographs were taken at 0 and 24 h following the initial scratch assay. The trans-well migration assay was used to confirm the anti-metastasis properties of SE-EA **(D)**, EGF-A **(E)**, and EGF-B **(F)** against LPS-ATP-induced A549 cells. The migrated cells were photographed under phase-contrast microscopy at 0 and 24 h and quantified using ImageJ software. Data are presented as mean ± S.D. of three independent experiments. **p < 0.01 and ***p < 0.001 indicating statistical significance compared to the LPS-ATP-induced control group.

Inflammatory response of lung cancer cells through the NLRP3 inflammasome pathway not only promotes cancer cell growth but also enhances cancer cell invasion and metastasis. Therefore, the effects of the SE-EA extract and its two active compounds, EGF-A and EGF-B, on the inhibition of lung cancer cell migration were investigated using would healing and trans-well assays in LPS-ATP-induced A549 lung cancer cells. The study found that LPS-ATP-induced A549 lung cancer cells had a higher migration capability than non-induced A549 cells, as shown in Figure 7. However, when treated with SE-EA extract, significant inhibition of cancer cell migration was observed in LPS-ATP-induced A549 lung cancer cells, as shown in Figures 7A, D. Moreover, the two active compounds, EGF-A and EGF-B, derived from SE-EA extract, also exhibited a significant inhibition of cancer cell migration in LPS-ATP-induced A549 lung cancer cells, as indicated in Figures 7B, C; Figures 7E, F.

### Effects of SE-EA and its active compounds (EGF-A and EGF-B) on LPS-ATP-induced A549 cells invasion

**FIGURE 8:**
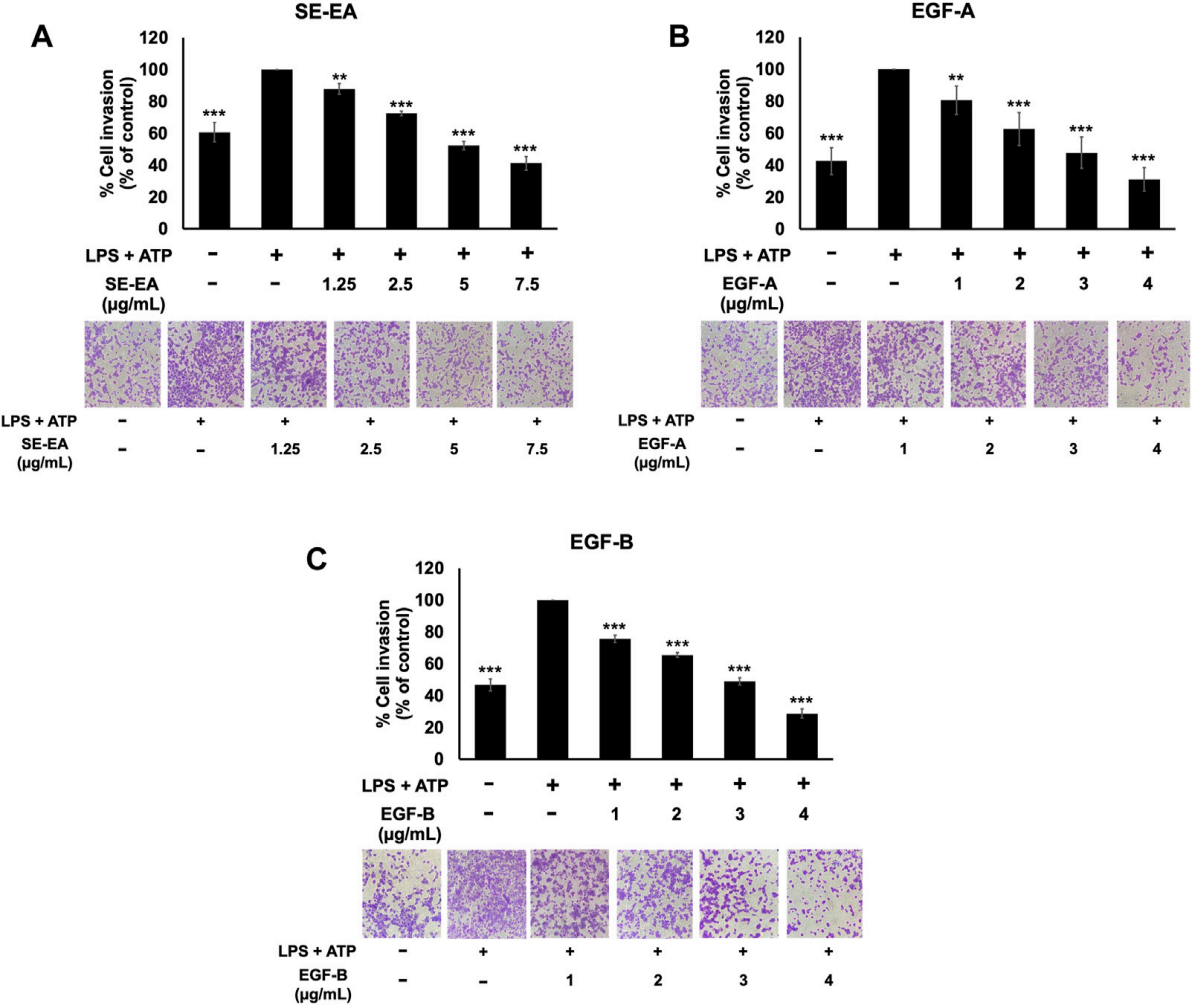
The effects of SE-EA and its active compounds (EGF-A and EGF-B) on the invasion of LPS-ATP-induced A549 cells. The anti-invasion effects of SE-EA **(A)** at concentrations ranging from 0-7.5 μg/mL, EGF-A **(B)** at concentrations of 0–4 μg/mL (0–9.42 µM), and EGF-B **(C)** at concentrations of 0–4 μg/mL (0–9.12 µM) on LPS-ATP-induced A549 cells were determined using a trans-well invasion assay. The migrated cells were imaged under phase-contrast microscopy at 0 and 24 h and quantified using ImageJ software. The data from three independent experiments are presented as mean ± S.D. The statistical significance was indicated as **p < 0.01 and ***p < 0.001, compared to the LPS-ATP-induced control group.

The effect of SE-EA and its two major compounds, EGF-A and EGF-B, on the invasion of A549 lung cancer cells were investigated using trans-well assay. The results showed that A549 lung cancer cells stimulated by LPS and ATP exhibited a higher invasion capability than non-stimulated A549 lung cancer cells, as shown in Figure 8. However, treatment with SE-EA extract and its active compounds, EGF-A and EGF-B, significantly inhibited the invasion of A549 lung cancer cells via the trans-well assay. Therefore, it can be concluded that the inflammatory response triggered by LPS-ATP via the NLRP3 inflammasome pathway enhances the invasive capability of A549 lung cancer cells. SE-EA extract and its active compounds, EGF-A and EGF-B can effectively suppress the invasion of A549 lung cancer cells stimulated by LPS-ATP via the NLRP3 inflammasome pathway.

### Effects of SE-EA and its active compounds (EGF-A and EGF-B) on inhibition of pro-inflammatory cytokines (IL-6, IL-1β, and IL-18) and NLRP3 gene expressions in LPS-ATP-induced A549 cells

**FIGURE 9:**
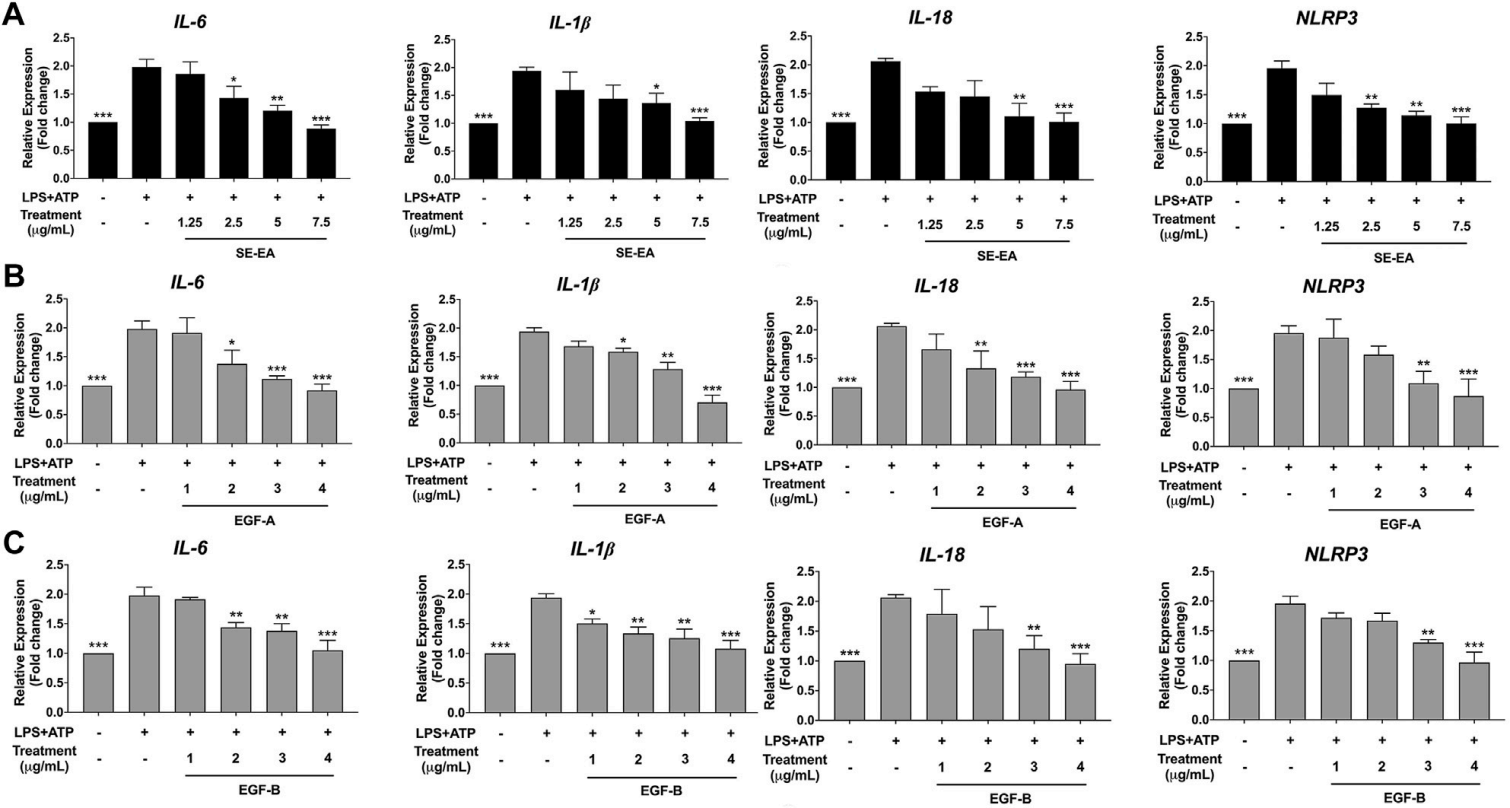
Inhibitory effects of SE-EA extract and its active compounds (EGF-A and EGF-B) on the NLRP3, IL-1β and IL-18 gene expression in LPS-ATP-induced A549 cells. A549 cells were treated with SE-EA extract **(A)** at concentrations of 0–7.5 μg/mL or active compounds, EGF-A **(B)** at concentrations of 0–4 μg/mL (0–9.42 µM) or EGF-B **(C)**, at concentrations of 0–4 μg/mL (0–9.12 µM) for 4 h. After treatment, the cells were induced by lipopolysaccharide (LPS) at a concentration of 1,000 ng/mL for 18 h, followed by adenosine triphosphate (ATP) at 5 nM for a further 6 h. The mRNA expressions of NLRP3, IL-6, IL-1β and IL-18 were determined using RT-qPCR. The LPS-ATP-induced A549 cells were used as a control group, and their expression levels were considered as 100%. The data are presented as mean ± S.D. values of three independent experiments. Statistical analysis was performed, *p < 0.05, **p < 0.01 and ***p < 0.001 were considered statistically significant compared to the LPS-ATP -induced control group.

The study aimed to investigate the mechanism of action of SE-EA extract, specifically EGE-A and EGF-B, in inhibiting the expression of pro-inflammatory cytokines IL-6 and inflammasome related cytokines IL-1β and IL-18, as well as the NLRP3 protein at the mRNA level using RT-qPCR. The results demonstrated that treatment of lung cancer A549 cells with LPS and ATP induced the expression of IL-6 and inflammasome-related cytokines IL-1β and IL-18, as well as increased NLRP3 gene expression. Conversely, treatment with SE-EA extract (Figure 9A), EGF-A (Figure 9B), and EGF-B (Figure 9C) significantly inhibited the expression of IL-6, IL-1β, IL-18, and NLRP3 genes in A549 cells stimulated with LPS and ATP in a concentration-dependent manner (*p < 0.05, **p < 0.01 and ***p < 0.001), compared to the LPS and ATP only group.

### Effects of SE-EA and its active compounds (EGF-A and EGF-B) on the NLRP3 inflammasome pathway in LPS-ATP-induced A549 cells

**FIGURE 10:**
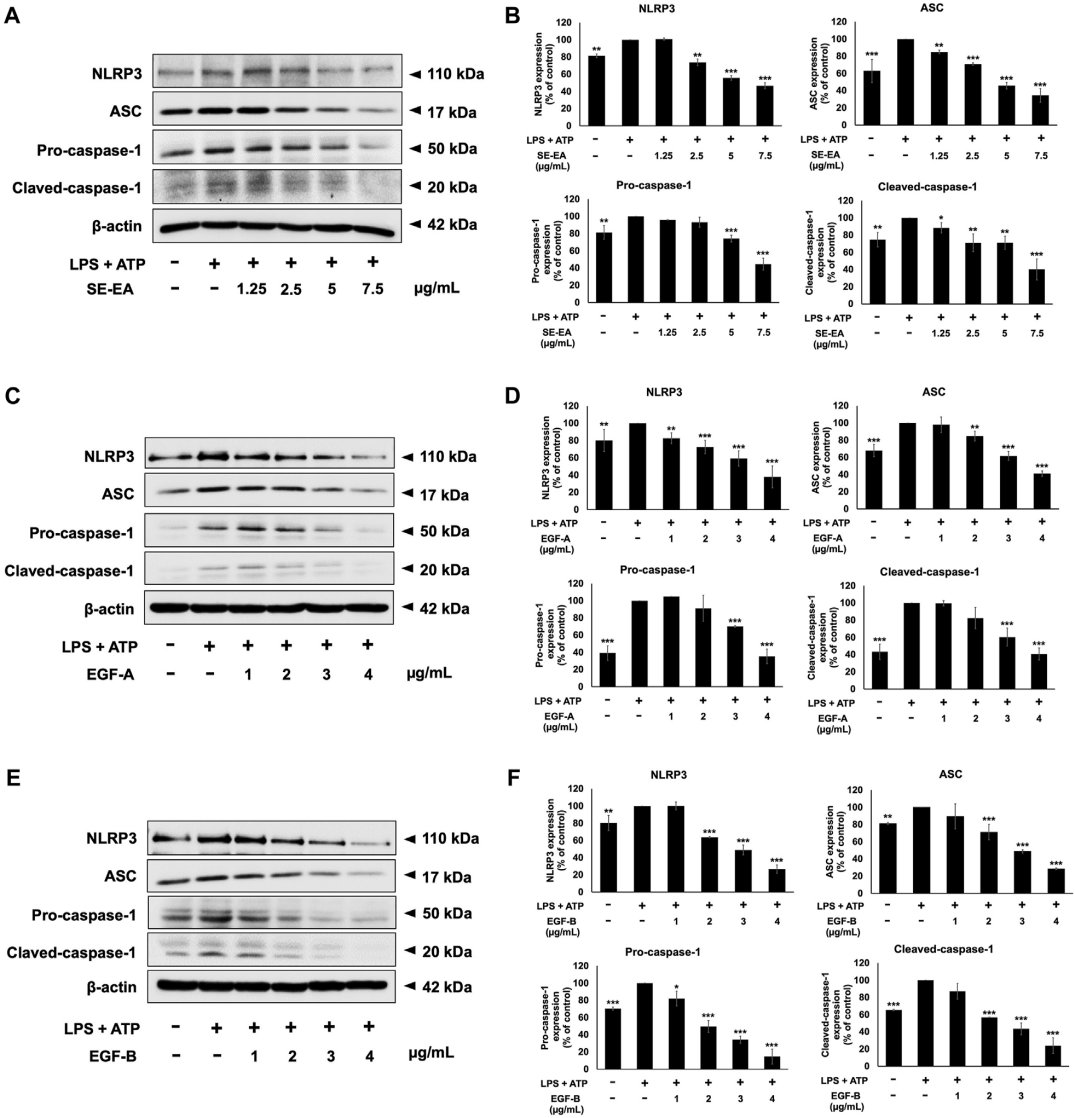
Inhibitory effects of *S. exigua* extracts and its active compounds (EGF-A and EGF-B) on the NLRP3 inflammasome pathway in LPS-ATP-induced A549 cells. A549 cells were treated with SE-EA **(A)** at concentrations ranging from 0-7.5 μg/mL or active compounds, EGF-A **(C)** at concentrations of 0–4 μg/mL (0–9.42 µM) or EGF-B **(E)**, at concentrations of 0–4 μg/mL (0–9.12 µM) for 4 h. The cells were induced by lipopolysaccharide (LPS) at a concentration of 1,000 ng/mL for 18 h, followed by adenosine triphosphate (ATP) at a concentration of 5 nM for a further 6 h. The inhibitory effects of SE-EA **(A, B)**, EGF-A **(C, D)**, and EGF-B **(E, F)** on the expression of NLRP3, ASC, and pro-caspase-1 and cleaved-caspase-1 proteins in A549 cells were measured using Western blot and band density measurements. The results were presented as a percentage of the LPS-ATP-induced A549 cells, where the control group was set to 100%. The data are presented as mean ± S.D. values of three independent experiments, with *p < 0.05, **p < 0.01 and ***p < 0.001 indicating statistical significance compared to the LPS-ATP-induced control group.

To induce inflammation, we treated A549 cells with LPS and ATP and compared these cells to a control group A549 cells that were not induced. It was found that, in A549 lung epithelial cells, the induction of inflammation using LPS and ATP resulted in elevated expression levels of NLRP3, ASC, pro-caspase-1 (p50), and cleaved-caspase-1 (p20) proteins when compared to non-induced control cells. Next, we performed Western blot analysis to evaluate the effects of SE-EA extract, EGF-A and EGF-B on the expression of these proteins in the LPS and ATP-induced A549 cells. Our results revealed that the SE-EA extract (Figures 10A, B), as well as EGF-A (Figures 10C, D) and EGF-B (Figures 10 E, F), significantly reduced the expression of NLRP3, ASC, pro-caspase-1 (p50), and cleaved-caspase-1 (p20) proteins in the LPS and ATP-induced A549 cells.

## Discussion

Research on the anticancer potential of *Sophora* species, including *Sophora flavescens* and *Sophora japonica*, has yielded promising results in inhibiting the growth of cancer cells (Krishna et al., 2012; Chen et al., 2013; He et al., 2015). For example, *Sophora flavescens*, commonly used in traditional Chinese medicine, has shown potential as an anticancer agent against lung cancer cells. Extracts from *Sophora flavescens* have demonstrated anti-proliferative effects and induced apoptosis in A549 and NCI-H226 cell lines (Chen et al., 2019). Similarly, extracts from *Sophora flavescens* and *Sophora japonica* have been investigated for their potential anticancer effects in MCF-7 breast cancer (Cao and He, 2020). Studies have isolated certain compounds from *Sophora* species that exhibit promising anticancer effects against liver cancer cells. These compounds, including flavonoids and alkaloids, have demonstrated inhibitory effects on cell proliferation, migration, and invasion in HepG2 cell lines (Lin et al., 2023). *Sophora japonica* and *Sophora subprostrata* have been studied for their potential anticancer effects on colorectal cancer cells. Extracts from these plants have shown cytotoxic activity against HT29 colorectal cancer cells and induced apoptosis in these cells (Fang et al., 2018; Chen et al., 2021). However, information specifically related to *Sophora exigua* and its anticancer potential is scarce. Hence, it is essential to study the anticancer properties of *S. exigua* plant extract and its bioactive compounds on the A549 non-small cell lung cancer cell line. Furthermore, investigating the underlying mechanisms involved in cancer progression is of utmost importance.

Exiguaflavanones, specifically flavanones, which are a subclass of flavonoids, are known for their diverse chemical structures and various biological activities (Ruangrungsi et al., 1992; Boozari et al., 2019; Abd-Alla et al., 2021). Flavanones have been studied for their potential health benefits and medicinal properties, including antioxidant, anti-inflammatory, antimicrobial, and anticancer activities (Tsuchiya et al., 1996; Fakhimi et al., 2006; Kaewdana et al., 2021). In the root of *Sophora exigua*, a plant known for its medicinal properties, two important flavanone compounds called Exiguaflavanone A (EGF-A) and exiguaflavanone (EGF-B) can be found. These compounds are obtained from the ethyl acetate fraction of the plant extract (SE-EA) and have yields of 3.94% and 5.96%, respectively. In the context of the NLRP3 inflammasome pathway, which plays a crucial role in the inflammatory response of lung cancer cells, we have expanded upon the mechanisms discussed in the references. Specifically, we have detailed the role of the NLRP3 inflammasome pathway in promoting cancer cell growth, invasion, and metastasis. This is achieved partly through the activation of the NLRP3 inflammasome pathway and the subsequent release of the IL-1β cytokine, as highlighted in previous studies (Fan et al., 2014; Wang et al., 2016b). Therefore, this study aimed to investigate the potential of the SE-EA extract and its active compounds, EGF-A and EGF-B, in inhibiting lung cancer cell proliferation, migration, and invasion. To induce inflammation in A549 non-small cell lung cancer cells, a combination of LPS, a bacterial inflammation-inducing agent, and ATP was used. The findings of our investigation revealed that SE-EA extract and its active compounds, EGF-A and EGF-B, effectively inhibited LPS- and ATP-induced proliferation of A549 cells. These compounds caused cell cycle arrest at the G1 phase and significantly downregulated the expression of cell cycle regulator proteins. Moreover, the SE-EA extract and its active compounds demonstrated a significant inhibitory effect on LPs-ATP-induced cell migration and invasion.

Inflammation is a complex process that involves the release of various cytokines and mediators. Notably, IL-6, Il-1β, and IL-18 are well-known pro-inflammatory cytokines that play a crucial role in the pathogenesis of many inflammatory diseases (Fenini et al., 2017). Previous studies have indicated that the activation of NLRP3 inflammasome can contribute to cancer progression by releasing inflammatory cytokines such as IL-1β and IL-18 (Vidal-Vanaclocha et al., 2000; Fabbi et al., 2015; Fenini et al., 2017). Our findings confirm the anti-inflammatory effects of SE-EA and its active compounds, EGF-A and EGF-B, by modulating the NLRP3 inflammasome pathway. These compounds effectively reduce cytokine release and downregulate key proteins involved in the NLRP3 inflammasome pathway, including NLRP3, ASC, pro-caspase-1 and cleaved-caspase-1. Furthermore, in our molecular investigations, we observed significant downregulation of mRNA expression of IL-1β, IL-18, and NLRP3 in LPS-ATP-induced A549 cells treated with SE-EA, EGF-A, and EGF-B, ultimately leading to a decrease in the release of inflammatory cytokines. The downregulation suggested that these compounds have the ability to suppress the transcriptional activity of these pro-inflammatory molecules. One potential mechanism through which this downregulation may occur is by inhibiting the activation of NF-kB. Several studies have reported that natural compounds can exert their anti-inflammatory effects by modulating NF-kB activity (Song et al., 2018; Semmarath et al., 2022; Lan et al., 2023). These compounds can interfere with the suppression of NF-kB nuclear translocation. By disrupting these steps, the compounds prevent the transactivation of NF-kB target genes involved in the inflammatory response. While the precise mechanism through which SE-EA, EGF-A, and EGF-B inhibit NF-kB activation and subsequently suppress transcriptional activity is not specified in this study, it is reasonable to propose NF-kB as a potential target. Further investigations, such as assessing NF-kB activation status would be necessary to confirm this hypothesis and provide more mechanistic insights into the transcriptional regulation.

Our study also contributes to the understanding of the role of inflammation in cancer progression. This observation aligns with previous research that has established a link between inflammation and cancer. For instance, the overexpression of NLRP3 inflammasome stimulated proliferation, migration, and invasion of esophageal squamous cell carcinoma (ESCC) *in vitro*. As evidence by the knocking down using NLRP3-siRNAs could result in the attenuation of ESCC metastatic potential. Hence, NLRP3 could be a promising new candidate of cancer targeted therapy (Yu et al., 2020). In the context of non-small cell lung cancer, the activation of the NLRP3 inflammasome pathway in response to LPS and ATP enhanced the cell proliferation and invasive capability of A549 cells. Furthermore, the caspase-1 inhibitor, Z-YVAD-FMK, suppressed LPS-ATP-induced cell migration and invasion by abolishing Akt phosphorylation and inhibiting ERK1/2 and CREB phosphorylation (Wang et al., 2016b). Our findings coincide with these previous reports, highlighting the potential influence of LPS and ATP-induced NLRP3 inflammasome pathway, consequently impacting cancer cell behavior.

Moreover, the previous reports emphasize the crucial role of NLRP3 inflammasome in regulating the proliferation, invasion, and migration of A549 cells. These studies underline the significance of chronic inflammation as a recognized risk factor for cancer development (Moossavi et al., 2018; Gouravani et al., 2020; Xu et al., 2021). Inflammasomes can contribute to tumor development by influencing host tumor immunity, promoting tumor cell proliferation and differentiation, and regulating the tumor microenvironment. Notably, the activation of the NLRP3 inflammasome in cancer-associated fibroblast (CAF) or CAF-derived IL-1β could facilitate lung cancer metastasis, as demonstrated by Moossavi et al., 2018; Gouravani et al., 2020. Nevertheless, it is essential to acknowledge the context-dependent and sometimes contrasting effects of the NLRP3 inflammasome and its product, IL-1β, on tumorigenesis depending on various cancer cell types. Evidence from documented studies and *in vivo* experiments suggests the activation of inflammasomes and related cytokines, such as IL-1β, in many human cancers. IL-1β plays a pivotal role in connecting innate and adaptive immune responses (Ghiringhelli et al., 2009), inducing the polarization of IFN-γ-secreting CD8^+^ T cells and triggering the formation of IL-17-producing γδT cells, which γδT cells have the ability to recognize tumor-associated antigens and perform anti-cancer activities (Ma et al., 2011). However, recent studies have identified IL-1β as a pro-cancer factor due to its immunosuppressive and chemo-resistant properties (Voronov et al., 2003). In the context of our study, we align with these recent findings, as we have observed that NLRP3 activation contributes to the progression of cancer cells in A549 lung cancer cells. Given the complex role of NLRP 3 inflammasome in the initiation and progression of neoplasia, the NLRP3 inflammasome and its associated pathways represent promising therapeutic targets for the prevention and treatment of lung cancer.

Our findings suggest that SE-EA extract and its active compounds, EGF-A and EGF-B, possess potential anti-cancer properties as they inhibit the invasion of A549 lung cancer cells stimulated by LPS and ATP via the NLRP3 inflammasome pathway. This discovery is significant since invasion is a crucial feature of cancer progression and inhibiting this process could potentially slow down or even halt cancer growth. However, before considering the clinical application of SE-EA extract and its active compounds, EGF-A and EGF-B, further studies are necessary to elucidate the underlying mechanisms of their metabolic pathways and to evaluate potential side effects in animal models. Understanding the precise mechanisms by which these compounds exert their anti-inflammatory and anti-cancer effects is essential for their therapeutic potential. Additionally, a comprehensive assessment of any possible adverse effects is crucial to ensure the safety and efficacy of these compounds. Animal models provide a valuable platform for investigating the pharmacokinetics, metabolic pathways, and toxicity profiles of SE-EA extract and its active compounds, thus aiding in determining their suitability for clinical use.

In conclusion, this study investigated the effects of *Sophora exigua* extract and its active compounds on the NLRP3 inflammasome pathway in non-small cell lung cancer (NSCLC). The results demonstrated that the extract, particularly the ethyl acetate fraction (SE-EA), exhibited significant anti-inflammatory properties and inhibited the production of pro-inflammatory cytokines in NSCLC cells. The active compounds, exiguaflavanone (EGF-A) and exiguaflavanone (EGF-B), were found to contribute to these effects. Moreover, SE-EA, EGF-A, and EGF-B showed anti-proliferative and anti-metastatic properties by affecting cell cycle progression, migration, and invasion in NSCLC cells. These findings suggest that targeting the NLRP3 inflammasome pathway could be a promising therapeutic approach for NSCLC treatment, providing insights into the development of novel anti-cancer therapeutics.

## Data Availability

The original contributions presented in the study are included in the article/Supplementary material, further inquiries can be directed to the corresponding author.

## References

[B1] Abd-Alla H. I. Souguir D. Radwan M. O. (2021). Genus Sophora: a comprehensive review on secondary chemical metabolites and their biological aspects from past achievements to future perspectives. Archives pharmacal Res. 44, 903–986. 10.1007/s12272-021-01354-2 PMC8671057 34907492

[B2] Alduais Y. Zhang H. Fan F. Chen J. Chen B. (2023). Non-small cell lung cancer (NSCLC): a review of risk factors, diagnosis, and treatment. Medicine 102, e32899. 10.1097/MD.0000000000032899 36827002 PMC11309591

[B3] Aly S. H. Elissawy A. M. Eldahshan O. A. Elshanawany M. A. Efferth T. Singab A. N. B. (2019). The pharmacology of the genus Sophora (Fabaceae): an updated review. Phytomedicine 64, 153070. 10.1016/j.phymed.2019.153070 31514082

[B4] Arjsri P. Srisawad K. Mapoung S. Semmarath W. Thippraphan P. Umsumarng S. (2022). Hesperetin from root extract of clerodendrum petasites s. Moore inhibits sars-Cov-2 spike protein S1 subunit-induced Nlrp3 inflammasome in A549 lung cells via modulation of the Akt/Mapk/Ap-1 pathway. Int. J. Mol. Sci. 23, 10346. 10.3390/ijms231810346 36142258 PMC9498987

[B5] Aryal S. Baniya M. K. Danekhu K. Kunwar P. Gurung R. Koirala N. (2019). Total phenolic content, flavonoid content and antioxidant potential of wild vegetables from Western Nepal. Plants 8, 96. 10.3390/plants8040096 30978964 PMC6524357

[B6] Boozari M. Soltani S. Iranshahi M. (2019). Biologically active prenylated flavonoids from the genus Sophora and their structure–activity relationship—a review. Phytotherapy Res. 33, 546–560. 10.1002/ptr.6265 30652369

[B7] Box J. (1983). Investigation of the Folin-Ciocalteau phenol reagent for the determination of polyphenolic substances in natural waters. Water Res. 17, 511–525. 10.1016/0043-1354(83)90111-2

[B8] Cao X. He Q. (2020). Anti-tumor activities of bioactive phytochemicals in Sophora flavescens for breast cancer. Cancer Manag. Res. 12, 1457–1467. 10.2147/CMAR.S243127 32161498 PMC7051174

[B9] Chen H. Yang J. Hao J. Lv Y. Chen L. Lin Q. (2019). A novel flavonoid kushenol Z from Sophora flavescens mediates mTOR pathway by inhibiting phosphodiesterase and Akt activity to induce apoptosis in non-small-cell lung cancer cells. Molecules 24, 4425. 10.3390/molecules24244425 31817093 PMC6943755

[B10] Chen H. Zhang J. Luo J. Lai F. Wang Z. Tong H. (2013). Antiangiogenic effects of oxymatrine on pancreatic cancer by inhibition of the NF-κB-mediated VEGF signaling pathway. Oncol. Rep. 30, 589–595. 10.3892/or.2013.2529 23754270

[B11] Chen M.-H. Gu Y.-Y. Zhang A. L. Sze D.M.-Y. Mo S.-L. May B. H. (2021). Biological effects and mechanisms of matrine and other constituents of Sophora flavescens in colorectal cancer. Pharmacol. Res. 171, 105778. 10.1016/j.phrs.2021.105778 34298110

[B12] Chittasupho C. Srisawad K. Arjsri P. Phongpradist R. Tingya W. Ampasavate C. (2023). Targeting spike glycoprotein S1 mediated by NLRP3 inflammasome machinery and the cytokine releases in A549 lung epithelial cells by nanocurcumin. Pharmaceuticals 16, 862. 10.3390/ph16060862 37375809 PMC10302714

[B13] Crowley L. C. Chojnowski G. Waterhouse N. J. (2016). Measuring the DNA content of cells in apoptosis and at different cell-cycle stages by propidium iodide staining and flow cytometry. Cold Spring Harb. Protoc. 2016, prot087247. 10.1101/pdb.prot087247 27698234

[B14] Dupaul-Chicoine J. Arabzadeh A. Dagenais M. Douglas T. Champagne C. Morizot A. (2015). The Nlrp3 inflammasome suppresses colorectal cancer metastatic growth in the liver by promoting natural killer cell tumoricidal activity. Immunity 43, 751–763. 10.1016/j.immuni.2015.08.013 26384545

[B15] Fabbi M. Carbotti G. Ferrini S. (2015). Context-dependent role of IL-18 in cancer biology and counter-regulation by IL-18BP. J. Leukoc. Biol. 97, 665–675. 10.1189/jlb.5RU0714-360RR 25548255

[B16] Fakhimi A. Iranshahi M. Emami S. A. Amin-Ar-Ramimeh E. Zarrini G. Shahverdi A. R. (2006). Sophoraflavanone G from Sophora pachycarpa enhanced the antibacterial activity of gentamycin against *Staphylococcus aureus* . Z. für Naturforsch. C 61, 769–772. 10.1515/znc-2006-9-1026 17137127

[B17] Fan S.-H. Wang Y.-Y. Lu J. Zheng Y.-L. Wu D.-M. Li M.-Q. (2014). Luteoloside suppresses proliferation and metastasis of hepatocellular carcinoma cells by inhibition of NLRP3 inflammasome. PloS one 9, e89961. 10.1371/journal.pone.0089961 24587153 PMC3935965

[B18] Fang R. Wu R. Zuo Q. Yin R. Zhang C. Wang C. (2018). Sophora flavescens containing-QYJD formula activates Nrf2 anti-oxidant response, blocks cellular transformation and protects against DSS-induced colitis in mouse model. Am. J. Chin. Med. 46, 1609–1623. 10.1142/S0192415X18500829 PMC8111688 30284461

[B19] Fenini G. Contassot E. French L. E. (2017). Potential of IL-1, IL-18 and inflammasome inhibition for the treatment of inflammatory skin diseases. Front. Pharmacol. 8, 278. 10.3389/fphar.2017.00278 28588486 PMC5438978

[B20] Franken N. A. Rodermond H. M. Stap J. Haveman J. Van Bree C. (2006). Clonogenic assay of cells *in vitro* . Nat. Protoc. 1, 2315–2319. 10.1038/nprot.2006.339 17406473

[B21] Ghiringhelli F. Apetoh L. Tesniere A. Aymeric L. Ma Y. Ortiz C. (2009). Activation of the NLRP3 inflammasome in dendritic cells induces IL-1beta-dependent adaptive immunity against tumors. Nat. Med. 15, 1170–1178. 10.1038/nm.2028 19767732

[B22] Gomes M. Teixeira A. L. Coelho A. Araujo A. Medeiros R. (2014). The role of inflammation in lung cancer. Inflamm. cancer 816, 1–23. 10.1007/978-3-0348-0837-8_1 24818717

[B23] Gouravani M. Khalili N. Razi S. Keshavarz-Fathi M. Khalili N. Rezaei N. (2020). The NLRP3 inflammasome: a therapeutic target for inflammation-associated cancers. Expert Rev. Clin. Immunol. 16, 175–187. 10.1080/1744666X.2020.1713755 31928260

[B24] He X. Fang J. Huang L. Wang J. Huang X. (2015). Sophora flavescens Ait.: traditional usage, phytochemistry and pharmacology of an important traditional Chinese medicine. J. Ethnopharmacol. 172, 10–29. 10.1016/j.jep.2015.06.010 26087234

[B25] He Y. Hara H. Núñez G. (2016). Mechanism and regulation of NLRP3 inflammasome activation. Trends Biochem. Sci. 41, 1012–1021. 10.1016/j.tibs.2016.09.002 27669650 PMC5123939

[B26] Huang C.-F. Chen L. Li Y.-C. Wu L. Yu G.-T. Zhang W.-F. (2017). NLRP3 inflammasome activation promotes inflammation-induced carcinogenesis in head and neck squamous cell carcinoma. J. Exp. Clin. Cancer Res. 36, 116–213. 10.1186/s13046-017-0589-y 28865486 PMC5581464

[B27] Huang J. Deng Y. Tin M. S. Lok V. Ngai C. H. Zhang L. (2022). Distribution, risk factors, and temporal trends for lung cancer incidence and mortality: a global analysis. Chest 161, 1101–1111. 10.1016/j.chest.2021.12.655 35026300

[B28] Huang L. Duan S. Shao H. Zhang A. Chen S. Zhang P. (2019). NLRP3 deletion inhibits inflammation-driven mouse lung tumorigenesis induced by benzo (a) pyrene and lipopolysaccharide. Respir. Res. 20, 20–29. 10.1186/s12931-019-0983-4 30696442 PMC6352353

[B29] Kabała-Dzik A. Rzepecka-Stojko A. Kubina R. Jastrzębska-Stojko Ż. Stojko R. Wojtyczka R. D. (2017). Migration rate inhibition of breast cancer cells treated by caffeic acid and caffeic acid phenethyl ester: an *in vitro* comparison study. Nutrients 9, 1144. 10.3390/nu9101144 29048370 PMC5691760

[B30] Kaewdana K. Chaniad P. Jariyapong P. Phuwajaroanpong A. Punsawad C. (2021). Antioxidant and antimalarial properties of Sophora exigua Craib. root extract in Plasmodium berghei-infected mice. Trop. Med. Health 49, 24–11. 10.1186/s41182-021-00314-2 33741053 PMC7980637

[B31] Ko J. Winslow M. M. Sage J. (2021). Mechanisms of small cell lung cancer metastasis. EMBO Mol. Med. 13, e13122. 10.15252/emmm.202013122 33296145 PMC7799359

[B32] Krishna P. M. Knv R. Banji D. (2012). A review on phytochemical, ethnomedical and pharmacological studies on genus Sophora, Fabaceae. Rev. Bras. Farmacogn. 22, 1145–1154. 10.1590/s0102-695x2012005000043

[B33] Lan C. Qian Y. Wang Y. Chen Y. Lin C. Zhang Y. (2023). The protective role of curcumin in human dental pulp stem cells stimulated by lipopolysaccharide via inhibiting NF-κB p65 phosphorylation to suppress NLRP3 inflammasome activation. Clin. Oral Investig. 27, 2875–2885. 10.1007/s00784-023-04885-8 36735089

[B34] Lee G. Walser T. C. Dubinett S. M. (2009). Chronic inflammation, chronic obstructive pulmonary disease, and lung cancer. Curr. Opin. Pulm. Med. 15, 303–307. 10.1097/MCP.0b013e32832c975a 19417670

[B35] Liang C.-C. Park A. Y. Guan J.-L. (2007). *In vitro* scratch assay: a convenient and inexpensive method for analysis of cell migration *in vitro* . Nat. Protoc. 2, 329–333. 10.1038/nprot.2007.30 17406593

[B36] Lin T.-Y. Tsai M.-C. Tu W. Yeh H.-C. Wang S.-C. Huang S.-P. (2021). Role of the NLRP3 inflammasome: insights into cancer hallmarks. Front. Immunol. 11, 610492. 10.3389/fimmu.2020.610492 33613533 PMC7886802

[B37] Lin Y. Chen X.-J. Li J.-J. He L. Yang Y.-R. Zhong F. (2023). A novel type lavandulyl flavonoid from Sophora flavescens as potential anti-hepatic injury agent that inhibit TLR2/NF-κB signaling pathway. J. Ethnopharmacol. 307, 116163. 10.1016/j.jep.2023.116163 36738945

[B38] Ma Y. Aymeric L. Locher C. Mattarollo S. R. Delahaye N. F. Pereira P. (2011). Contribution of IL-17-producing gamma delta T cells to the efficacy of anticancer chemotherapy. J. Exp. Med. 208, 491–503. 10.1084/jem.20100269 21383056 PMC3058575

[B39] Moossavi M. Parsamanesh N. Bahrami A. Atkin S. L. Sahebkar A. (2018). Role of the NLRP3 inflammasome in cancer. Mol. cancer 17, 158–213. 10.1186/s12943-018-0900-3 30447690 PMC6240225

[B40] Noreen H. Semmar N. Farman M. Mccullagh J. S. (2017). Measurement of total phenolic content and antioxidant activity of aerial parts of medicinal plant Coronopus didymus. Asian Pac. J. Trop. Med. 10, 792–801. 10.1016/j.apjtm.2017.07.024 28942828

[B41] Olcum M. Tufekci K. U. Durur D. Y. Tastan B. Gokbayrak I. N. Genc K. (2021). Ethyl pyruvate attenuates microglial NLRP3 inflammasome activation via inhibition of HMGB1/NF-κB/miR-223 signaling. Antioxidants 10, 745. 10.3390/antiox10050745 34066647 PMC8151120

[B42] Pękal A. Pyrzynska K. (2014). Evaluation of aluminium complexation reaction for flavonoid content assay. Food Anal. Methods 7, 1776–1782. 10.1007/s12161-014-9814-x

[B43] Pitchakarn P. Suzuki S. Ogawa K. Pompimon W. Takahashi S. Asamoto M. (2012). Kuguacin J, a triterpeniod from Momordica charantia leaf, modulates the progression of androgen-independent human prostate cancer cell line, PC3. Food Chem. Toxicol. 50, 840–847. 10.1016/j.fct.2012.01.009 22266361

[B44] Ruangrungsi N. Iinuma M. Tanaka T. Ohyama M. Yokoyama J. Mizuno M. (1992). Three flavanones with a lavandulyl group in the roots of Sophora exigua. Phytochemistry 31, 999–1001. 10.1016/0031-9422(92)80202-p

[B45] Semmarath W. Mapoung S. Umsumarng S. Arjsri P. Srisawad K. Thippraphan P. (2022). Cyanidin-3-O-glucoside and peonidin-3-O-glucoside-rich fraction of black rice germ and bran suppresses inflammatory responses from SARS-CoV-2 spike glycoprotein S1-induction *in vitro* in A549 lung cells and THP-1 macrophages via inhibition of the NLRP3 inflammasome pathway. Nutrients 14, 2738. 10.3390/nu14132738 35807916 PMC9268823

[B46] Semmarath W. Srisawad K. Arjsri P. Umsumarng S. Yodkeeree S. Jamjod S. (2023). Protective effects of proanthocyanidin-rich fraction from red rice germ and bran on lung cell inflammation via inhibition of NF-κB/NLRP3 inflammasome pathway. Nutrients 15, 3793. 10.3390/nu15173793 37686825 PMC10490275

[B47] Sodde V. K. Lobo R. Kumar N. Maheshwari R. Shreedhara C. (2015). Cytotoxic activity of Macrosolen parasiticus (L.) Danser on the growth of breast cancer cell line (MCF-7). Pharmacogn. Mag. 11, S156–S160. 10.4103/0973-1296.157719 26109761 PMC4461955

[B48] Song D. Zhao J. Deng W. Liao Y. Hong X. Hou J. (2018). Tannic acid inhibits NLRP3 inflammasome-mediated IL-1β production via blocking NF-κB signaling in macrophages. Biochem. biophysical Res. Commun. 503, 3078–3085. 10.1016/j.bbrc.2018.08.096 30126633

[B49] Stares M. Ding T.-E. Stratton C. Thomson F. Baxter M. Cagney H. (2022). Biomarkers of systemic inflammation predict survival with first-line immune checkpoint inhibitors in non-small-cell lung cancer. ESMO open 7, 100445. 10.1016/j.esmoop.2022.100445 35398717 PMC9058907

[B50] Suarez-Arnedo A. Figueroa F. T. Clavijo C. Arbeláez P. Cruz J. C. Muñoz-Camargo C. (2020). An image J plugin for the high throughput image analysis of *in vitro* scratch wound healing assays. PloS one 15, e0232565. 10.1371/journal.pone.0232565 32722676 PMC7386569

[B51] Sukkasem K. (2015). Biological activities of Thai traditional remedy called Kheaw-Hom and its plant ingredients. Bangkok, Thailand: Thammasat University.

[B52] Sukkasem K. Panthong S. Itharat A. (2016). Antimicrobial activities of Thai traditional remedy “kheaw-hom” and its plant ingredients for skin infection treatment in chickenpox. J. Med. Assoc. Thail. Chotmaihet Thangphaet 99, 116–123. 29926689

[B53] Tsuchiya H. Sato M. Miyazaki T. Fujiwara S. Tanigaki S. Ohyama M. (1996). Comparative study on the antibacterial activity of phytochemical flavanones against methicillin-resistant *Staphylococcus aureus* . J. Ethnopharmacol. 50, 27–34. 10.1016/0378-8741(96)85514-0 8778504

[B54] Vichai V. Kirtikara K. (2006). Sulforhodamine B colorimetric assay for cytotoxicity screening. Nat. Protoc. 1, 1112–1116. 10.1038/nprot.2006.179 17406391

[B55] Vidal-Vanaclocha F. Fantuzzi G. Mendoza L. Fuentes A. M. Anasagasti M. J. Martín J. (2000). IL-18 regulates IL-1beta-dependent hepatic melanoma metastasis via vascular cell adhesion molecule-1. Proc. Natl. Acad. Sci. 97, 734–739. 10.1073/pnas.97.2.734 10639148 PMC15399

[B56] Voronov E. Shouval D. S. Krelin Y. Cagnano E. Benharroch D. Iwakura Y. (2003). IL-1 is required for tumor invasiveness and angiogenesis. Proc. Natl. Acad. Sci. 100, 2645–2650. 10.1073/pnas.0437939100 12598651 PMC151394

[B57] Wang H. Chen L. Zhang L. Gao X. Wang Y. Weiwei T. (2016a). Protective effect of sophoraflavanone G on streptozotocin (STZ)-induced inflammation in diabetic rats. Biomed. Pharmacother. 84, 1617–1622. 10.1016/j.biopha.2016.10.113 27832995

[B58] Wang H. Kong H. Zeng X. Liu W. Wang Z. Yan X. (2016b). Activation of NLRP3 inflammasome enhances the proliferation and migration of A549 lung cancer cells. Oncol. Rep. 35, 2053–2064. 10.3892/or.2016.4569 26782741

[B59] Wu J. Lin Z. (2022). Non-small cell lung cancer targeted therapy: drugs and mechanisms of drug resistance. Int. J. Mol. Sci. 23, 15056. 10.3390/ijms232315056 36499382 PMC9738331

[B60] Xu Z. Wang H. Qin Z. Zhao F. Zhou L. Xu L. (2021). NLRP3 inflammasome promoted the malignant progression of prostate cancer via the activation of caspase-1. Cell death Discov. 7, 399. 10.1038/s41420-021-00766-9 34930938 PMC8688424

[B61] Yao M. Fan X. Yuan B. Takagi N. Liu S. Han X. (2019). Berberine inhibits NLRP3 Inflammasome pathway in human triple-negative breast cancer MDA-MB-231 cell. BMC Complementary Altern. Med. 19, 216–311. 10.1186/s12906-019-2615-4 PMC6694465 31412862

[B62] Yu S. Yin J. J. Miao J. X. Li S. G. Huang C. Z. Huang N. (2020). Activation of NLRP3 inflammasome promotes the proliferation and migration of esophageal squamous cell carcinoma. Oncol. Rep. 43, 1113–1124. 10.3892/or.2020.7493 32323780 PMC7057919

[B63] Zappa C. Mousa S. A. (2016). Non-small cell lung cancer: current treatment and future advances. Transl. Lung Cancer Res. 5, 288–300. 10.21037/tlcr.2016.06.07 27413711 PMC4931124

